# The Egyptian Collaborative Cardiac Genomics (ECCO-GEN) Project: defining a healthy volunteer cohort

**DOI:** 10.1038/s41525-020-00153-w

**Published:** 2020-10-23

**Authors:** Yasmine Aguib, Mona Allouba, Alaa Afify, Sarah Halawa, Mohamed El-Khatib, Marina Sous, Aya Galal, Eslam Abdelrahman, Nairouz Shehata, Amr El Sawy, Mohamed Elmaghawry, Shehab Anwer, Omnia Kamel, Wesam El Mozy, Hadir Khedr, Ahmed Kharabish, Nagwa Thabet, Pantazis I. Theotokis, Rachel Buchan, Risha Govind, Nicola Whiffin, Roddy Walsh, Heba Aguib, Ahmed Elguindy, Declan P. O’Regan, Stuart A. Cook, Paul J. Barton, James S. Ware, Magdi Yacoub

**Affiliations:** 1 Aswan Heart Centre, Aswan, Egypt; 2 National Heart and Lung Institute, Imperial College London, London, UK; 3 Cardiovascular Research Centre, Royal Brompton and Harefield NHS Foundation Trust, London, UK; 4 Biotechnology Graduate Program, American University in Cairo, New Cairo, Egypt; 5 University Heart Center Zürich, Zürich, Switzerland; 6 Radiology department, Kasr Alainy School of Medicine, Cairo University, Cairo, Egypt; 7 MRC London Institute of Medical Sciences, Imperial College London, London, UK; 8 Department of Experimental Cardiology, Amsterdam Cardiovascular Sciences, Amsterdam UMC, Amsterdam, Netherlands; 9 National Heart Centre Singapore, Singapore, Singapore; 10 Duke-National University of Singapore, Singapore, Singapore; 11 Harefield Heart Science Centre, Harefield, Middlesex UK

**Keywords:** Cardiomyopathies, Personalized medicine

## Abstract

The integration of comprehensive genomic and phenotypic data from diverse ethnic populations offers unprecedented opportunities toward advancements in precision medicine and novel diagnostic technologies. Current reference genomic databases are not representative of the global human population, making variant interpretation challenging, especially in underrepresented populations, such as the North African population. To address this, the Egyptian Collaborative Cardiac Genomics (ECCO-GEN) Project launched a study comprising 1000 individuals free of cardiovascular disease (CVD). Here, we present the first 391 Egyptian healthy volunteers recruited to establish a pilot phenotyped control cohort. All individuals underwent detailed clinical investigation, including cardiac magnetic resonance imaging (MRI), and were sequenced using a targeted panel of 174 genes with reported roles in inherited cardiac conditions. We identified 1262 variants in 27 cardiomyopathy genes of which 15.1% were not captured in current global and regional genetic reference databases (here: gnomAD and Great Middle Eastern Variome). The ECCO-GEN project aims at defining the genetic landscape of an understudied population and providing individual-level genetic and phenotypic data to support future studies in CVD and population genetics.

## Introduction

Cardiovascular disease (CVD) is a major cause of death and disability worldwide^1,2^, and its prevalence continues to increase in low- and middle-income countries toward epidemic proportions^3,4^. Effective tailored strategies for the prevention and treatment of disease depend on thorough understanding of the molecular determinants and mechanisms involved, which may differ between specific populations. The rapid evolution of genomic and personalized precision medicine offers unprecedented opportunities in this regard^5,6^. These, however, are critically dependent on defining the genetic landscape of different populations, their individuals and the relation to their dynamic phenotype^7,8^. In-depth information is lacking in populations with a high disease burden, and yet they continue to be grossly understudied^9–13^. Notable initiatives and studies have recently begun in the Middle East and North Africa (MENA) region aiming at data collection and harmonization, paving the way for large-scale genomic studies^14,15^. To directly address these issues, the Egyptian Collaborative Cardiac Genomics (ECCO-GEN) Project is recruiting 1000 Egyptian healthy volunteers (EHVols) from the general population, who consent to be recalled to future research and are simultaneously establishing a regional biobank that hosts a broad range of biological samples for prospective studies. Participants are fully phenotyped with respect to cardiovascular health. The full dataset of 1000 EHVols will aid in distinguishing between incidental and medically actionable variants, and thus enhance diagnostic and treatment strategies. Here, we describe the protocol of the study and examine background genetic variation in genes previously shown to be involved in inherited cardiac conditions (ICCs), with a special focus on hypertrophic cardiomyopathy (HCM) and dilated cardiomyopathy (DCM).

## Results

### Characteristics of the EHVol Study Population

**Fig. 1:**
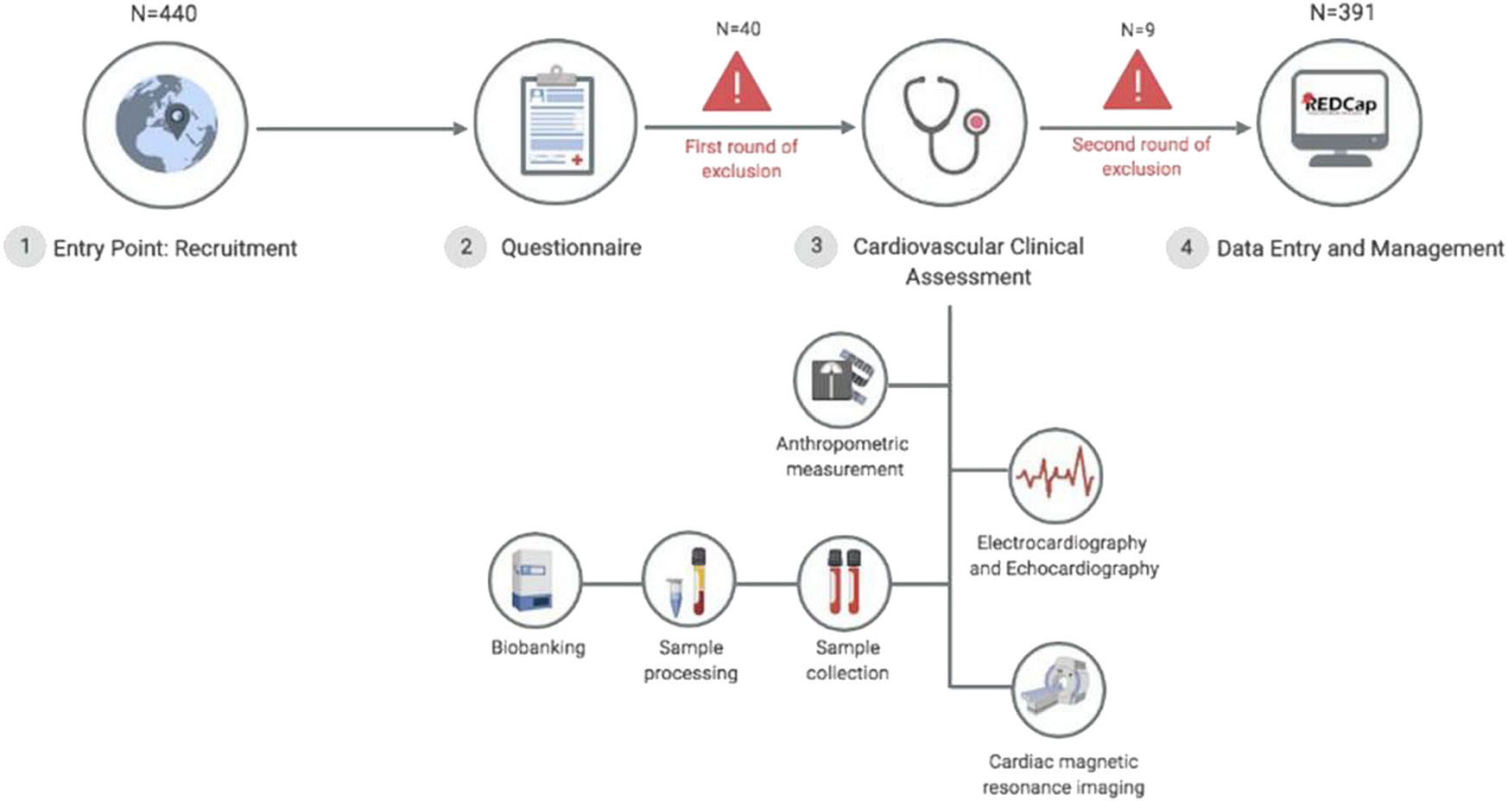
Workflow of the ECCO-GEN EHVol study. Study participants (1) were recruited from the general population via announcements (brochures, flyers, and public events); (2) completed a questionnaire providing demographic data, family, and clinical history; and (3) underwent detailed cardiovascular clinical assessment and blood sampling. (4) All data were recorded and managed in a local REDCap database. Rounds of exclusion: first round of exclusion is based on the basis of demographic and general health questionnaires as described under “study protocol and data collection”. A second round of exclusion was based on detailed cardiovascular phenotyping as described under “cardiovascular phenotyping” in the “Methods” section. ECCO-GEN The Egyptian Collaborative Cardiac Genomics, EHVol Egyptian healthy volunteers. Created with BioRender.

We initially screened 440 self-reported healthy individuals of whom 11% were excluded for not meeting the first round of inclusion criteria (see “Methods”, Supplementary Table 1 and Fig. 1). The remaining healthy individuals; the EHVols (*n* = 391), were genetically characterized using the ICC gene panel^16^. The general and cardiac characteristics of the study population are summarized in Table 1. The study population comprised 166 females (42.5%) and 225 males (57.5%). The mean age (years) was 33.2 (SD 9.5).

**Table 1:** Summary of general self-reported and cardiac characteristics of the EHVol (*n* = 391).

General characteristics	EHVols *n* (%)	Available data (*n*)
Age (years) Mean (SD)	33.2 (9.5)	391
Gender		391
Males	225 (57.5%)	
Females	166 (42.5%)	
Offspring of consanguineous marriage (self-reported)	64 (16.5%)	388
Smoking		390
Former smoker	11 (2.8%)	
Current smoker	83 (21.3%)	
Nonsmoker	296 (75.9%)	
Alcohol		389
1 glass/week	3 (0.8%)	
2–3 glasses/week	1 (0.3%)	
Unspecified	3 (0.8%)	
Family history of CVD	64 (16.4%)	391
BMI		352
<18.5	14 (4.0%)	
18.5–25	127 (36.1%)	
25–30	128 (36.4%)	
>30	83 (23.6%)	
Heart rate (beat/min) mean (SD)	75.7 (13.1%)	319

Cardiac characteristics	Mean (standard deviation)	Available data (*n*)
ECG variables		340
PR interval (ms)	147.3 (21.8)	
QRS duration (ms)	91.1 (47.8)	
QTc interval (ms)	401.8 (44.4)	
CMR variables		391
LVEF (%)	62.9 (5.4)	
LVMi (g/m^2^)	46.3 (13.8)	
LVESVi (ml/m^2^)	27.7 (6.9)	
LVEDVi (ml/m^2^)	74.5 (11.8)	

*EHVols* Egyptian healthy volunteers.

### Representation of EHVol genetic variation in reference genetic datasets

**Fig. 2:**
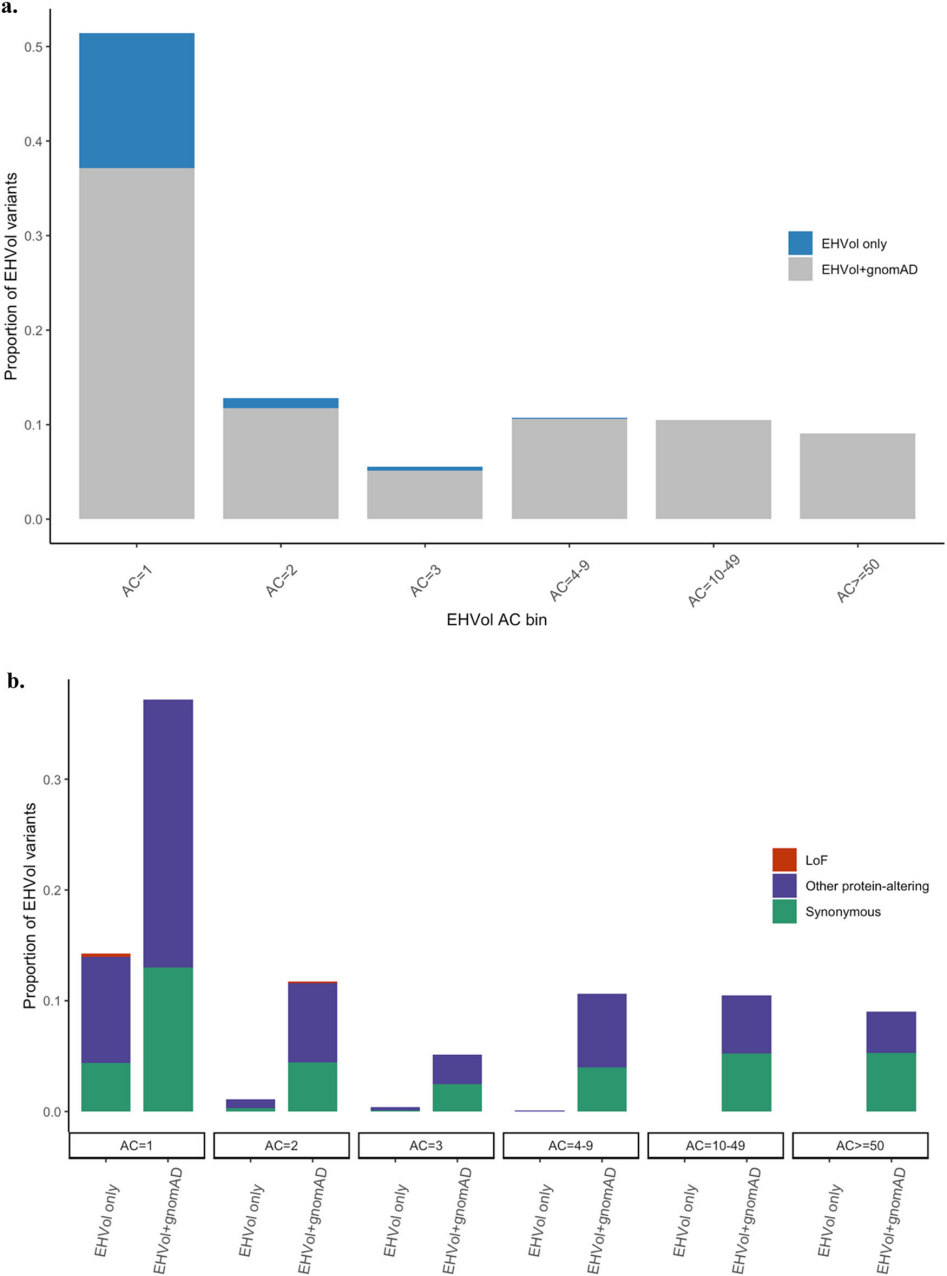
All of the variants absent from gnomAD were rare in the EHVol cohort (seen in <1% of the cohort). **a** Bar graph showing the distribution of observed ACs (binned) of variants identified in the EHVol cohort. The proportion of variants that were present in gnomAD (all populations) is shown in gray and the proportion of variants that were only present in our cohort is represented in blue. **b** Bar graph showing the classification of the EHVol variants in each AC bin for variants that were captured in gnomAD (EHVol + gnomAD) and variants that were absent from gnomAD (EHVol only). Variants included in this analysis were collated by LoF, other protein-altering, and synonymous variants (color-coded in figure). EHVol Egyptian healthy volunteer, AC: allele count.

We assessed the representation of the EHVol cohort in the Genome Aggregation Database (gnomAD) (https://gnomad.broadinstitute.org; version v2.1.1). GnomAD is the largest population reference dataset to date. It comprises genetic data from unrelated individuals from five main populations: African/African American, Admixed American, East Asian, Non-Finnish European, and South Asian, while data from the MENA region was lacking in the dataset. Individuals were sequenced by whole-exome sequencing (WES; *n* = 125,748) and whole-genome sequencing (WGS; *n* = 15,708)^17^ (see “Methods”). A total of 1262 variants were identified in the EHVol cohort in validated cardiomyopathy (CM) genes (see Supplementary Table 2 for transcript details), as well as genes implicated in syndromes that may present with isolated left ventricular hypertrophy (LVH)^18^ (Supplementary Table 3; see “Methods”). Variants identified in the EHVol cohort were categorized into three classes: “loss-of-function” (LoF; predicted truncating variants, i.e., frameshift, splice acceptor, splice donor, and nonsense), “synonymous”, and “other protein altering” (i.e., missense, inframe deletion, inframe insertion,…etc.). We calculated the proportion of EHVol variants present in gnomAD within each corresponding observed allele count (AC) bin. Of the 1262 EHVol CM variants, 1062 (84.2%) were captured in gnomAD, including all the variants that were common (>1%) in the EHVol cohort (*n* = 283; Fig. 2a and Table 2). The majority of non-gnomAD variants (EHVol only; *n* = 200) were singletons (AC bin 1) and were mostly classified as “other protein altering” (Fig. 2a, b). Among these singletons, LoF represented 2% of variants absent from gnomAD, as compared to those present (0.2%), indicating that underrepresented populations can potentially contribute to pathogenicity interpretation. Differences in sequencing coverage between WES data (gnomAD) and gene panel data (EHVol) are expected; thus, we assessed the coverage of the positions of non-gnomAD variants (*n* = 200) in gnomAD (Supplementary Fig. 2). We then compared the proportion of non-gnomAD variants in our cohort to the proportion of non-gnomAD variants in an ethnic control group that is generally represented in gnomAD, the Caucasian healthy volunteers (CHVols) cohort (see Methods). The CHVols comprised 1028 CHVols who were also sequenced using the Trusight Cardio Sequencing Kit for ICC^16,19^. As expected, the proportion of EHVol non-gnomAD variants was significantly higher (*p* value = 1.9e−06) than that of the CHVol cohort (15.9% and 10.3%, respectively; Table 2). The proportion of controls with LoF variants in CM genes was comparable between the EHVol (0.48%) and CHVol (0.3%) cohorts.

**Table 2:** Proportion of non-gnomAD variants in the EHVol and CHVol cohorts.

Cohort	Cohort size	Total no. of variants in CM and syndromic genes	Total no. of non-gnomAD variants^a^	Prop. of non- gnomAD variants	Total no. of non-GME variants	Prop. of non-GME variants
EHVols	391	1262	200	15.9%*	524	41.5%
CHVols	1028	2344	241	10.3%*	2339	99.8%

*EHVols* Egyptian healthy volunteers, *CHVols* Caucasian healthy volunteers.

*Fisher’s exact test (two-sided) *p* value = 1.943e−06.

^a^Variants found in gnomAD in low quality were included in the analysis.

In addition, we assessed the representation of the EHVol variants in another reference dataset, the Great Middle Eastern Variome (GME; *n* = 1111), as it includes WES data from the ethnically matched North African subpopulations^14^. Only ten non-gnomAD EHVol variants (0.79%) were exclusively present in the GME dataset and 524 (41.5%) variants were absent from it (Table 2). EHVol variants that were only present in GME were rare (AC = 1 or 2) and were mostly “other protein-altering” variants (data not shown).

### High frequency of rare variation in the EHVol cohort

**Fig. 3:**
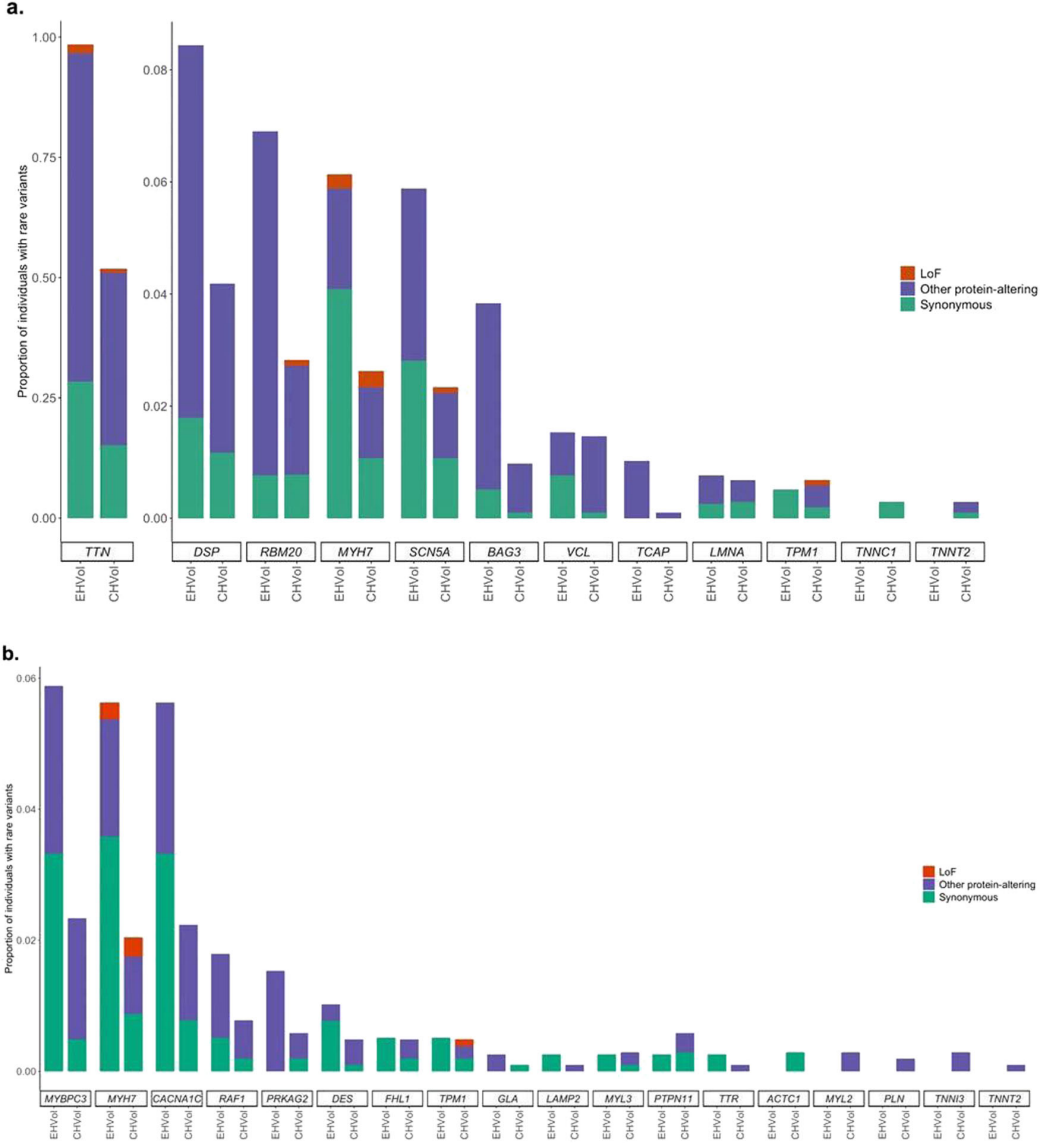
Rare variation in CM genes in EHVol and CHVol controls. Bar charts represent the proportion of individuals with rare variants in **a** DCM and **b** HCM, and LVH syndromic genes. The first and second bars represent the CHVol and EHVol cohorts, respectively. Variants are collated by variant class (LoF, synonymous, and other protein altering). CM cardiomyopathy, EHVol Egyptian healthy volunteer, CHVol Caucasian healthy volunteers, DCM dilated cardiomyopathy, HCM hypertrophic cardiomyopathy, LVH left ventricular hypertrophy.

We compared the frequency of rare protein-altering variation in the EHVol cohort with the CHVol cohort, in order to study the background genetic variation in CM-associated genes (see “Methods”). Since we had individual-level data for both control cohorts, we were able to report and compare the proportion of individuals with rare variants in DCM (gnomAD filtering allele frequency (FAF) popmax ≤ 8.4 × 10^−5^ (ref. ^20^)) and HCM (gnomAD FAFpopmax ≤ 4.0 × 10^−5^ (ref. ^20^)) genes (Fig. 3). Individuals were counted more than once if they carried more than one variant type in a gene. In both cohorts, *TTN*, *DSP*, *RBM20*, *MYH7*, and *SCN5A* accounted for the majority of rare variation in DCM genes (Fig. 3a). The predominant HCM genes *MYBPC3*, *MYH7*, and the syndromic LVH gene *CACNA1C* accounted for the majority of rare variation in both cohorts (Fig. 3b).

### Comparison of the distribution of rare *TTN* and *MYH7* variants between the different control cohorts

**Fig. 4:**
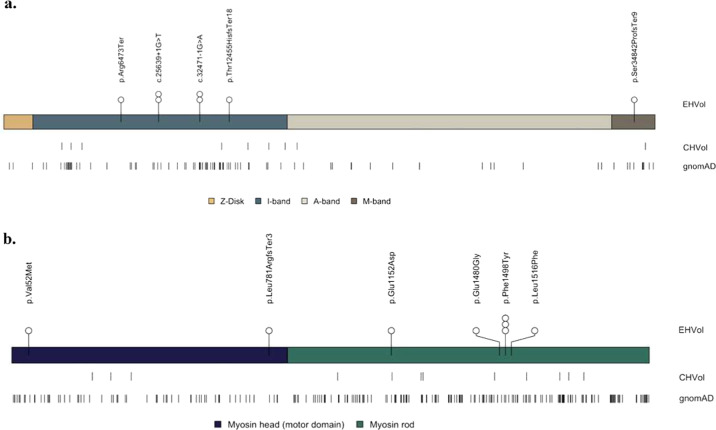
Distribution of rare *TTN LoF* and *MYH7* missense and LoF variants in the EHVol, CHVol, and gnomAD cohorts. Variant distribution is shown relative to a schematic representation of the **a** *TTN* protein, with sarcomere regions delimited and the **b** *MYH7* protein with myosin domains delimited. The number of circles represent the number of individuals carrying the rare variant. EHVol Egyptian healthy volunteer, CHVol Caucasian healthy volunteers, *TTN* (ENST00000589042); *MYH7* (ENST00000355349).

We investigated the distribution of rare variants in *TTN* (ENST00000589042) and *MYH7* (ENST00000355349), as different positional distributions of rare variants between cases and controls have been previously reported^21,22^. The analysis was restricted to rare LoF variants in *TTN*, and both missense and LoF variants in *MYH7* (Fig. 4), as they may be putative pathogenic^23,24^. Across all non-diseased cohorts, the majority of *TTN* variants were located in exons that are not constitutively expressed in the myocardium (Fig. 4a). One LoF variant identified in an EHVol (0.26%; p.Ser34842ProfsTer9) and six LoF variants identified in CHVols (0.58%; p.Glu35478Ter, p.Gln16235Ter, p.Gln15575Ter, c.44015-1 G > T, p.Pro4353GlnfsTer14, and p.Gln3243Ter) resided in cardiac constitutive exons, which would have been interpreted as “likely pathogenic” (LP) were they identified in individuals presenting with DCM^25^. Such variants are of unknown penetrance and frequently observed in individuals with no disease^26,27^. The LoF variant p.Ser34842ProfsTer9 identified in the EHVol cohort, is absent from both gnomAD and CHVol controls. According to the guidelines by the American College of Medical Genetics and the American Association of Pathology (ACMG/AMP), the p.Ser34842ProfsTer9 should be classified as LP. This classification is based on the activation of two ACMG/AMP rules, which are: the strong PVS1 “absent in population databases” rule and moderate PM2 rule “predicted null variant in a gene where LOF is a known mechanism of disease”^28^. However, the individual harboring this variant, who was clinically assessed at the age of 33, did not show a clinical DCM phenotype. Regular clinical follow-up will be performed for this individual. Moreover, across all populations, the majority of rare variants in *MYH7* (popmax FAF ≤ 4.0 × 10^−5^) were located outside of the functional domains that typically harbor HCM-associated variants (protein residues: 167–931; EHVol 4 (66.7%); CHVol 9 (75%); gnomAD 163 (71.5%); Fig. 4b). In addition, the *MYH7* LoF variant (p.Leu781ArgfsTer3) identified in one EHVol control did not fulfill the ACMG/AMP criteria for a “benign” or “pathogenic” classification, and thus its default ACMG/AMP classification was “variant of uncertain significance”.

## Discussion

The availability of large-scale population genetic databases, such as gnomAD, has opened up new possibilities in identifying putative disease-causing variants. Current datasets, however, do not adequately represent genetic variation in the MENA region, which hinders accurate variant interpretation in the respective populations. Several recent studies address this shortcoming. Scott et al., for instance, studied rare genetic variation of the GME region, including North East- and North West Africa^14^. Individuals recruited to the aforementioned study were from the general population (self-reported healthy volunteers), and thus may not necessarily be free of disease. To our knowledge, our study represents the first in the region to include high-coverage sequencing data from a clinically phenotyped healthy cohort. All EHVol controls were consented for recall for future studies to allow us to assess the effect of particular genotypes of interest on the clinical phenotype.

EHVol variants that were represented in both reference datasets comprised 57.69% (*n* = 728) of the variants. Given that our population is represented in the GME dataset, we expected more variants to be captured in it. This highlights the underrepresentation of the Egyptian population in current global and regional reference genetic databases. Overall, the frequency of rare variants in CM-associated genes was higher among EHVols compared to CHVols. However, it is important to note here that “rare” was defined using the frequency reported in gnomAD. Hence, observing this excess of variation in a CM free cohort likely reports variants that are more common in the Egyptian population rather than a higher burden of disease-related rare variation. This is supported by the phenotyping data. Our findings emphasize the importance of examining the rate of background rare variation in CM genes in ethnically distinct cohorts to allow us to discriminate the subset of variants that may be associated with an increased risk of disease.

Although the cohort size reported is still relatively small, this study provides preliminary insights into genetic variation of the underrepresented multi-ethnic Egyptian population. Expanding the ECCO-GEN project and further integration of ancestry-specific genetic and phenotypic data will deepen our understanding of African diversity, support variant interpretation and variant discovery. This will provide important information on disease-related mechanisms and a vital tool for precision medicine in ethnically related populations and in Africa.

## Methods

### Study protocol and data collection

This is a population-based study to recruit 1000 EHVols from across Egypt. All participants provided informed consent, and the study was reviewed and approved by the local research ethics committee (20151125MYFAHC_Hvol). Here, we report data from an initial cohort of 391 volunteers recruited from December 2015 to June 2018 via national advertisement (brochures, flyers, and public events) and assessed at the Aswan Heart Centre (AHC). A paper-based questionnaire was conducted to gather information regarding demographics, health status, smoking and drinking habits, medical and surgical history, medication, consanguineous marriage (defined as self-reported first-cousin marriage) and family history (defined as having one or more first-degree relatives with self-reported heart disease). In the first round of exclusion, individuals were excluded if they met any of the following criteria: <18 years of age, non-Egyptian nationality, pregnancy, presentation with known cardiovascular or collagen vascular disease, communication difficulties or contraindication to CMR. Data were entered into a Research Electronic Data Capture (REDCap) database and was stored, along with all appropriate documentation, on an access-controlled server^29^.

### Cardiovascular phenotyping

Eligible individuals underwent detailed cardiovascular phenotyping, including clinical examination, 12-lead electrocardiogram and CMR (Fig. 1). CMR was performed with a 1.5 T scanner (Siemens Magnetom Aera, Erlangen, Germany) using retrospective ECG triggering to capture the heart during the cardiac cycle. Steady-state free precession end-expiratory breath-hold cine images were acquired in the short axis orientation covering the whole heart. Standard parameters were repetition, time/echo time 3.6/1.8 ms; sense factor 2, flip angle, 60°; section thickness, 8 mm; no slice gap, matrix, 160 × 256; field of view, 300 mm; pixel size, 1.6 × 1.6 mm; number of phases 30 and phase percentage 67%. Phase-contrast images were acquired at different aortic levels for flow mapping. At the same acquisition levels, 4D flow was performed to assess flow patterns. T1 mapping was performed on base, mid and apical heart levels for fibrosis assessment. The 3D tagging acquisition was done at base, mid, and apical levels for strain assessment. Detailed structural and functional analysis on the CMR acquisitions was performed retrospectively using dedicated post processing and in-house software. Following phenotyping, a second round of exclusions, on the basis of specific cardiovascular diagnostic criteria, was applied (Table 3). Isolated apical noncompaction with normal ECG and normal CMR was not excluded.

**Table 3:** Second round of exclusion.

Second round of exclusion criteria
Electrocardiogram (ECG) findings:
• Abnormal QT interval
• High-degree atrioventricular block
• Type I Brugada ECG
• Atrial fibrillation
• Non-sustained VT
Cardiac magnetic resonance (CMR) findings^a^:
• Left ventricular mass index (LVM_i_) ≥ 99.5 g/m^2^ in males and 85.5 g/m^2^ in females
• Left ventricular end-systolic volume (LVESV_i_) ≥ 44.5 ml/m^2^ in males and 40.1 ml/m^2^ in females
• Ejection fraction < 52.5% in males and 53.2% and in females
• LV wall thickness ≥ 15 mm (measured at any segment)
• Non-compacted-to-compacted myocardium thickness of ≥2.3:1 affecting mid and/or basal segment (i.e., not limited to the apex/apical segments)

Participants were excluded if any of these findings were identified.

^a^Cutoff thresholds were determined as three standard deviations from the mean to be more inclusive of extremes, especially in the absence of normal CMR reference values specific to the Egyptian population^37^.

### Sample collection and biobanking

A total of 20 ml whole venous blood were withdrawn from each participant for biomarker measurement (hemoglobin A1c and troponin I), and biobanking of serum, plasma, and DNA. For DNA extraction, blood samples were transferred to K3EDTA tubes to avoid clotting. Blood samples were stored at 4 °C (max. 5 days) prior to DNA extraction. DNA was extracted using Wizard® Genomic DNA Purification Kit (Promega, Catalog No. A1620) according to manufacturer’s instructions. Concentration of 1 μl DNA sample was determined using the NanoDrop 2000 (Thermo Scientific) spectrophotometer. A 260/280 and 260/230 nm ratios were used to assess the DNA quality. All samples were stored centrally in the AHC Biobank.

### DNA sequencing

EHVols were sequenced with the Illumina Miseq and Nextseq platforms using the Trusight Cardio Sequencing Kit (Illumina, Catalog No. FC-141-1010 (MiSeq) and FC-141-1011 (NextSeq)) comprising 174 genes with reported roles in ICCs^16^. Sequencing was performed following the manufacturer’s protocol. The concentration and quality of the DNA libraries were evaluated using Qubit (Invitrogen) and TapeStation 4200 (Agilent Technologies).

### Bioinformatics pipeline

Raw data were subject to quality control using FastQC v0.10.1 (ref. ^30^) and low-quality reads were trimmed via prinseq-lite v0.20.4 (ref. ^31^). Trimmed reads were then mapped to hg19 (ref. ^32^) using the Burrows–Wheeler Aligner v0.7.10-r789 (ref. ^33^). After alignment, removal of duplicate reads was performed using picard v1.117 (“Picard Tools,” n.d.). GATK v3.2-2-gec30cee^34^ was then used for realignment of insertions or deletions (indels), as well as base quality score recalibration. Variant calling was performed using GATK’s HaplotypeCaller. Joint genotyping was performed with GATK v4.0.8.1 and hard filters were applied based on GATK best practices workflow for germline short variant discovery^34^. Only variants marked as “PASS”, with a quality of depth score ≥4 and an allelic balance ≥ 0.20 were retained. We attained high coverage of the target region (>99%) at ≥20× read depth (Supplementary Fig. 1). The Ensembl Variant Effect Predictor^35^ was used for variant annotation.

### Data analysis

#### Analysis of background genetic variation in selected cardiomyopathy genes

To examine the background genetic variation among genes involved in inherited CMs; specifically, DCM and HCM, we selected genes that were recently validated (Supplementary Table 2). The following 12 DCM genes were analyzed^36^: *BAG3*, *DSP, LMNA*, *MYH7*, *RBM20*, *SCN5A*, *TCAP*, *TNNC1*, *TNNT2*, *TPM1*, *TTN*, and *VCL*. For HCM genes, the following eight genes were analyzed: *MYBPC3, MYH7, TNNT2, TNNI3, TPM1, ACTC1, MYL2, MYL3* as they have been reported to be definitively associated with the disease^18^. In addition, nine genes associated with syndromes that include cardiac hypertrophy and can present with isolated LVH (*CACNA1C*, *DES*, *FHL1*, *GLA*, *LAMP2*, *PRKAG2*, *PTPN11*, *RAF1*, and *TTR)* were analyzed, and also *PLN*, which is associated with a variable cardiomyopathic phenotype^18^.

#### Comparison of genetic variation between EHVol and gnomAD controls

We downloaded WES data from the gnomAD database^17^ (https://gnomad.broadinstitute.org; version v2.1.1) and used it to assess genetic variation in CM genes between the EHVol and gnomAD controls. We restricted our analysis to protein-coding regions only. Identified variants were categorized into three classes: LoF (predicted truncating variants, i.e., frameshift, splice acceptor, splice donor, and nonsense), synonymous and other protein-altering (missense, inframe deletion, inframe insertion,…etc.).

#### Comparison of rare variation between EHVol and CHVol controls

The CHVols were recruited prospectively via advertisement for the UK Digital Heart Project at Imperial College London. All volunteers underwent CMR to confirm the absence of cardiac disease. The frequency of rare variation in the selected CM genes was compared between the EHVol and CHVol cohorts. The threshold maximum credible population allele frequency (MAF) for putative disease-causing variants in DCM (≤8.4 × 10^−5^) and HCM (≤4.0 × 10^−5^) were defined using the statistical framework developed by Whiffin et al.^20^ as denoted by the following equation^19^: MAF = (disease prevalence × maximum allelic contribution (i.e., genetic heterogeneity)/penetrance). The MAF was then compared with the FAF available in gnomAD, which is the precomputed AF threshold calculated for all variants in gnomAD, as described in ref. ^20^. For an HCM/DCM variant to be considered potentially disease causing, its MAF should be lower than the popmax FAF. The “popmax FAF” is the highest calculated AF among the five main gnomAD subpopulations. The frequency of rare variants per gene in the EHVol/CHVol cohorts was calculated by counting the number of rare variants per gene and dividing this by the cohort size (EHVols: *n* = 391, CHVols: *n* = 1028).

### Reporting summary

Further information on research design is available in the Nature Research Reporting Summary linked to this article.

## Supplementary information

Supplementary Information

Reporting Summary

## Data Availability

Sequencing data (ICC panel) from this study were submitted to the European Genome-phenome Archive (EGA), which is hosted by The European Bioinformatics Institute (EBI) and the Centre for Genomic Regulation (CRG) under the accession code: EGAD00001006160. All other data generated or analyzed during this study are included in this published article (and its Supplementary information files).

## Code availability

Processing of raw sequence files, variant calling, variant annotation, and all other analyses described in this study were performed using available and open source software, as described in the “Methods” section. No custom code was therefore used, apart from simple scripts integrating commands for such programs.
