# Supplementary material for: The Egyptian Collaborative Cardiac Genomics (ECCO-GEN) Project: defining a healthy volunteer cohort
Source: NPJ Genom Med. 2020 Oct 23;5:46. doi: 10.1038/s41525-020-00153-w (PMC7584615; doi:10.1038/s41525-020-00153-w)
Supplement: Supplementary file 1 — Supplementary Information [file 41525_2020_153_MOESM1_ESM.pdf]

Supplementary Table 1: Detected ECG, CMR findings and genetic results of the excluded individuals (n=9) based on the second round of exclusion

Age	Gender	ECG findings	CMR findings	Genetic Results
25	Male	First-degree heart block	Dilated LV (LVESVI = 50 ml/m2)	No rare variation in DCM/HCM genes
25	Male	None	Dilated LV (LVESVI = 49 ml/m2)	No rare variation in DCM/HCM genes
25	Male	None	Dilated LV with impaired systolic function (LVESVI = 50 ml/m2, EF= 51%)	No rare variation in DCM/HCM genes
26	Male	None	Dilated LV (LVESVI = 47 ml/m2	Non-gnomAD frameshift VUS in DSP (p.Ile2622ThrfsTer16)
30	Male	None	Dilated LV (LVESVI =46 ml/m2)	No rare variation in DCM/HCM genes
31	Male	Left anterior hemiblock, incomplete right bundle branch block and first-degree heart block	None	No rare variation in DCM/HCM genes
34	Male	None	Dilated LV (LVESVI = 54 ml/m2)	No rare variation in DCM/HCM genes
41	Male	None	Impaired LV systolic function (EF = 50%)	No rare variation in DCM/HCM genes
47	Male	None	Dilated LV with impaired systolic function (LVESVI = 75 ml/m2 EF = 35~%)	No rare variation in DCM/HCM genes

Supplementary Table 2: Genes and transcripts analysed in this study

Gene	Ensembl transcript	Refseq transcript	Cardiomyopathy
ACTC1	ENST00000290378	NM_005159	HCM
BAG3	ENST00000369085	NM_004281	DCM
CACNA1C	ENST00000399655	NM_000719	syndromic gene (associated with isolated LVH)
DES	ENST00000373960	NM_001927	syndromic gene (associated with isolated LVH)
DSP	ENST00000379802	NM_004415	DCM
FHL1	ENST00000370690	NM_001449	syndromic gene (associated with isolated LVH)
GLA	ENST00000218516	NM_000169	syndromic gene (associated with isolated LVH)

LAMP2	ENST00000200639	NM_002294	syndromic gene (associated with isolated LVH)
LMNA	ENST00000368300	NM_170707	DCM
MYBPC3	ENST00000545968	NM_000256	HCM
MYH7	ENST00000355349	NM_000257	HCM & DCM
MYL2	ENST00000228841	NM_000432	HCM
MYL3	ENST00000395869	NM_000258	HCM
PLN	ENST00000357525	NM_002667	HCM
PRKAG2	ENST00000287878	NM_016203	syndromic gene (associated with isolated LVH)
PTPN11	ENST00000351677	NM_002834	syndromic gene (associated with isolated LVH)
RAF1	ENST00000251849	NM_002880	syndromic gene (associated with isolated LVH)
RBM20	ENST00000369519	NM_001134363	DCM
SCN5A	ENST00000333535	NM_198056	DCM
TCAP	ENST00000309889	NM_003673	DCM
TNNC1	ENST00000232975	NM_003280	DCM
TNNI3	ENST00000344887	NM_000363	HCM
TNNT2	ENST00000367318	NM_001001430	HCM & DCM
TPM1	ENST00000403994	NM_001018005	HCM & DCM
TTN	ENST00000589042	NM_001267550	DCM
TTR	ENST00000237014	NM_000371	syndromic gene (associated with isolated LVH)
VCL	ENST00000211998	NM_014000	DCM

Supplementary Table 3: Variants identified in the EHVol cohort. See Supplemental Table 2 for the transcripts analysed for each gene.

**Cardiomyopathy**	**Gene**	**cdna variant**	**protein variant**	**Consequence**	**EHVol count**	**EHVol frequency (n=391)**	**gnomAD popmax FAF**
DCM	LMNA	c.659G>A	p.Arg220His	missense_variant	1	0.002557545	1.10E-05
DCM	LMNA	c.1189C>T	p.Arg397Cys	missense_variant	1	0.002557545	1.10E-05
DCM & HCM	TNNT2	c.758A>G	p.Lys253Arg	missense_variant	49	0.125319693	0.148523
DCM & HCM	TNNT2	c.83C>T	p.Ala28Val	missense_variant	1	0.002557545	0.000563
DCM	BAG3	c.169G>A	p.Glu57Lys	missense_variant	1	0.002557545	0
DCM	BAG3	c.187C>G	p.Pro63Ala	missense_variant	1	0.002557545	0.010563
DCM	BAG3	c.249C>A	p.His83Gln	missense_variant	1	0.002557545	0.009116
DCM	BAG3	c.337G>A	p.Val113Ile	missense_variant	1	0.002557545	1.20E-05
DCM	BAG3	c.418G>A	p.Gly140Ser	missense_variant	2	0.00511509	1.20E-05
DCM	BAG3	c.451T>C	p.Cys151Arg	missense_variant	90	0.230179028	0.215415
DCM	BAG3	c.695A>C	p.His232Pro	missense_variant	1	0.002557545	0
DCM	BAG3	c.794C>T	p.Pro265Leu	missense_variant	1	0.002557545	0
DCM	BAG3	c.821C>T	p.Ser274Leu	missense_variant	1	0.002557545	0.00077
DCM	BAG3	c.1138C>T	p.Pro380Ser	missense_variant	1	0.002557545	0.010503
DCM	BAG3	c.1220C>T	p.Pro407Leu	missense_variant	57	0.145780051	0.259528
DCM	BAG3	c.1423G>A	p.Asp475Asn	missense_variant	6	0.015345269	8.40E-05
DCM	BAG3	c.1447G>A	p.Gly483Ser	missense_variant	1	0.002557545	0
DCM	BAG3	c.1634C>G	p.Pro545Arg	missense_variant	1	0.002557545	0.000109
DCM	RBM20	c.70T>A	p.Cys24Ser	missense_variant	1	0.002557545	0
DCM	RBM20	c.206T>C	p.Leu69Pro	missense_variant	1	0.002557545	0
DCM	RBM20	c.349G>A	p.Ala117Thr	missense_variant	1	0.002557545	0
DCM	RBM20	c.419C>T	p.Pro140Leu	missense_variant	5	0.012787724	0
DCM	RBM20	c.485C>T	p.Thr162Ile	missense_variant	2	0.00511509	0
DCM	RBM20	c.517C>A	p.Pro173Thr	missense_variant	11	0.028132992	0.074854
DCM	RBM20	c.680G>T	p.Gly227Val	missense_variant	8	0.020460358	0.001556

DCM	RBM20	c.695G>A	p.Gly232Asp	missense_variant	20	0.051150895	0.070814
DCM	RBM20	c.1067C>T	p.Thr356Ile	missense_variant	1	0.002557545	0
DCM	RBM20	c.1093G>A	p.Gly365Arg	missense_variant	1	0.002557545	0.004603
DCM	RBM20	c.1139G>A	p.Arg380Gln	missense_variant	2	0.00511509	3.20E-05
DCM	RBM20	c.1144G>T	p.Ala382Ser	missense_variant	1	0.002557545	0
DCM	RBM20	c.1841A>T	p.His614Leu	missense_variant	1	0.002557545	0
DCM	RBM20	c.1938G>C	p.Arg646Ser	missense_variant	1	0.002557545	0
DCM	RBM20	c.2131C>T	p.Arg711Cys	missense_variant	1	0.002557545	0
DCM	RBM20	c.2303G>C	p.Trp768Ser	missense_variant	391	1	0.993012
DCM	RBM20	c.2318A>G	p.Lys773Arg	missense_variant	4	0.010230179	0.008207
DCM	RBM20	c.2507A>G	p.Glu836Gly	missense_variant	1	0.002557545	0
DCM	RBM20	c.2627C>A	p.Ser876Tyr	missense_variant	1	0.002557545	0
DCM	RBM20	c.3076G>A	p.Glu1026Lys	missense_variant	4	0.010230179	5.90E-05
DCM	RBM20	c.3373G>A	p.Glu1125Lys	missense_variant	7	0.017902813	0.004619
DCM	RBM20	c.3661C>T	p.Arg1221Cys	missense_variant	1	0.002557545	6.00E-06
DCM	RBM20	c.3667G>C	p.Glu1223Gln	missense_variant	358	0.915601023	0.86018
DCM	VCL	c.587A>C	p.Asn196Thr	missense_variant	1	0.002557545	0
DCM	VCL	c.2852C>T	p.Pro951Leu	missense_variant	1	0.002557545	0
DCM	VCL	c.3267G>C	p.Glu1089Asp	missense_variant	1	0.002557545	4.40E-05
HCM	MYBPC3	c.3535G>A	p.Glu1179Lys	missense_variant	1	0.002557545	0.000988
HCM	MYBPC3	c.3373G>A	p.Val1125Met	missense_variant	2	0.00511509	1.20E-05
HCM	MYBPC3	c.3224C>G	p.Thr1075Ser	missense_variant	1	0.002557545	0
HCM	MYBPC3	c.3107G>A	p.Arg1036His	missense_variant	1	0.002557545	6.10E-05
HCM	MYBPC3	c.3106C>T	p.Arg1036Cys	missense_variant	2	0.00511509	0.004656
HCM	MYBPC3	c.2989T>C	p.Phe997Leu	missense_variant	1	0.002557545	0
HCM	MYBPC3	c.2956_2958delAAG	p.Lys986del	inframe_deletion	1	0.002557545	0

HCM	MYBPC3	c.2908C>T	p.Arg970Trp	missense_variant ,splice_region_variant	1	0.002557545	0
HCM	MYBPC3	c.2882C>T	p.Pro961Leu	missense_variant	1	0.002557545	9.70E-05
HCM	MYBPC3	c.2345A>G	p.Asn782Ser	missense_variant	1	0.002557545	0
HCM	MYBPC3	c.2210C>T	p.Thr737Met	missense_variant	1	0.002557545	7.50E-05
HCM	MYBPC3	c.1855G>A	p.Glu619Lys	missense_variant	3	0.007672634	0.000317
HCM	MYBPC3	c.1564G>A	p.Ala522Thr	missense_variant	1	0.002557545	0.003087
HCM	MYBPC3	c.1519G>A	p.Gly507Arg	missense_variant	1	0.002557545	0.004424
HCM	MYBPC3	c.1321G>A	p.Glu441Lys	missense_variant	7	0.017902813	0.000216
HCM	MYBPC3	c.1144C>T	p.Arg382Trp	missense_variant	7	0.017902813	0.041035
HCM	MYBPC3	c.977G>A	p.Arg326Gln	missense_variant	3	0.007672634	0.005274
HCM	MYBPC3	c.833G>A	p.Gly278Glu	missense_variant	2	0.00511509	0.012769
HCM	MYBPC3	c.787G>A	p.Gly263Arg	missense_variant	1	0.002557545	0.000249
HCM	MYBPC3	c.706A>G	p.Ser236Gly	missense_variant	86	0.219948849	0.128629
HCM	MYBPC3	c.649A>G	p.Ser217Gly	missense_variant	2	0.00511509	0.009787
HCM	MYBPC3	c.646G>A	p.Ala216Thr	missense_variant	1	0.002557545	0.002011
HCM	MYBPC3	c.565G>A	p.Val189Ile	missense_variant	18	0.046035806	0.003394
HCM	MYBPC3	c.495G>C	p.Glu165Asp	missense_variant	1	0.002557545	0.00017
HCM	MYBPC3	c.472G>A	p.Val158Met	missense_variant	49	0.125319693	0.091891
HCM	MYBPC3	c.305C>T	p.Pro102Leu	missense_variant	2	0.00511509	3.70E-05
HCM	MYBPC3	c.194C>T	p.Thr65Met	missense_variant	1	0.002557545	0
HCM	MYBPC3	c.67G>A	p.Ala23Thr	missense_variant	1	0.002557545	8.80E-05
syndromic gene (associated with isolated LVH)	CACNA1C	c.109G>A	p.Gly37Arg	missense_variant	1	0.002557545	0.004922
syndromic gene (associated with isolated LVH)	CACNA1C	c.1468G>A	p.Gly490Arg	missense_variant	1	0.002557545	0.000233

syndromic gene (associated with isolated LVH)	CACNA1C	c.3368G>A	p.Arg1123His	missense_variant	1	0.002557545	1.70E-05
syndromic gene (associated with isolated LVH)	CACNA1C	c.3533A>G	p.Lys1178Arg	missense_variant	1	0.002557545	0
syndromic gene (associated with isolated LVH)	CACNA1C	c.4942G>A	p.Ala1648Thr	missense_variant	1	0.002557545	8.90E-05
syndromic gene (associated with isolated LVH)	CACNA1C	c.5023G>A	p.Ala1675Thr	missense_variant	1	0.002557545	0.000153
syndromic gene (associated with isolated LVH)	CACNA1C	c.5109C>G	p.Phe1703Leu	missense_variant	1	0.002557545	0
syndromic gene (associated with isolated LVH)	CACNA1C	c.5150C>G	p.Ala1717Gly	missense_variant	4	0.010230179	0.000937
syndromic gene (associated with isolated LVH)	CACNA1C	c.5198C>T	p.Ala1733Val	missense_variant	2	0.00511509	1.00E-05
syndromic gene (associated with isolated LVH)	CACNA1C	c.5284G>A	p.Gly1762Ser	missense_variant	1	0.002557545	0
syndromic gene (associated with isolated LVH)	CACNA1C	c.5360C>T	p.Thr1787Met	missense_variant	3	0.007672634	0.011507
syndromic gene (associated with isolated LVH)	CACNA1C	c.5383G>A	p.Gly1795Arg	missense_variant	8	0.020460358	0.050466
syndromic gene (associated with isolated LVH)	CACNA1C	c.5534C>T	p.Thr1845Met	missense_variant	1	0.002557545	0

syndromic gene (associated with isolated LVH)	CACNA1C	c.5917C>T	p.Arg1973Trp	missense_variant	2	0.00511509	0
syndromic gene (associated with isolated LVH)	CACNA1C	c.5918G>A	p.Arg1973Gln	missense_variant	3	0.007672634	0.014183
syndromic gene (associated with isolated LVH)	CACNA1C	c.6059G>A	p.Ser2020Asn	missense_variant	2	0.00511509	5.90E-05
syndromic gene (associated with isolated LVH)	PTPN11	c.893A>G	p.Asn298Ser	missense_variant	1	0.002557545	5.60E-05
DCM & HCM	MYH7	c.4548G>C	p.Leu1516Phe	missense_variant	1	0.002557545	0
DCM & HCM	MYH7	c.4493T>A	p.Phe1498Tyr	missense_variant	3	0.007672634	0
DCM & HCM	MYH7	c.4472C>G	p.Ser1491Cys	missense_variant	11	0.028132992	0.012294
DCM & HCM	MYH7	c.4439A>G	p.Glu1480Gly	missense_variant	1	0.002557545	0
DCM & HCM	MYH7	c.3456G>C	p.Glu1152Asp	missense_variant	1	0.002557545	0
DCM & HCM	MYH7	c.968T>C	p.Ile323Thr	missense_variant	1	0.002557545	0.000267
DCM & HCM	MYH7	c.154G>A	p.Val52Met	missense_variant	1	0.002557545	0
DCM	TCAP	c.113G>T	p.Cys38Phe	missense_variant ,splice_region_variant	1	0.002557545	0.000159
DCM	TCAP	c.208C>T	p.Arg70Trp	missense_variant	2	0.00511509	2.40E-05
DCM	TCAP	c.301G>A	p.Ala101Thr	missense_variant	1	0.002557545	0
DCM	TCAP	c.316C>T	p.Arg106Cys	missense_variant	4	0.010230179	0.14892
DCM	TCAP	c.353C>T	p.Ala118Val	missense_variant	1	0.002557545	0.00215
DCM	TCAP	c.422C>T	p.Pro141Leu	missense_variant	1	0.002557545	0
syndromic gene (associated with isolated LVH)	TTR	c.76G>A	p.Gly26Ser	missense_variant	1	0.002557545	0.07181

syndromic gene (associated with isolated LVH)	TTR	c.328C>A	p.His110Asn	missense_variant	8	0.020460358	0.000373
syndromic gene (associated with isolated LVH)	TTR	c.424G>A	p.Val142Ile	missense_variant	1	0.002557545	0.014106
syndromic gene (associated with isolated LVH)	DES	c.166G>C	p.Val56Leu	missense_variant	1	0.002557545	0.00037
syndromic gene (associated with isolated LVH)	DES	c.170C>T	p.Ser57Leu	missense_variant	1	0.002557545	0.00062
syndromic gene (associated with isolated LVH)	DES	c.216C>A	p.Ser72Arg	missense_variant	1	0.002557545	0.00012
syndromic gene (associated with isolated LVH)	DES	c.638C>T	p.Ala213Val	missense_variant ,splice_region_va riant	3	0.007672634	0.014612
syndromic gene (associated with isolated LVH)	DES	c.934G>A	p.Asp312Asn	missense_variant	1	0.002557545	0.001487
syndromic gene (associated with isolated LVH)	DES	c.935A>C	p.Asp312Ala	missense_variant	1	0.002557545	0.002947
syndromic gene (associated with isolated LVH)	DES	c.1048C>T	p.Arg350Trp	missense_variant	1	0.002557545	7.00E-06
syndromic gene (associated with isolated LVH)	DES	c.1375G>A	p.Val459Ile	missense_variant	4	0.010230179	0.030072
DCM	TTN	c.107696T>C	p.Ile35899Thr	missense_variant	1	0.002557545	5.40E-05

DCM	TTN	c.107302G>A	p.Asp35768As n	missense_variant	1	0.002557545	0
DCM	TTN	c.107267T>C	p.Val35756Ala	missense_variant	75	0.191815857	0.171908
DCM	TTN	c.107182G>A	p.Glu35728Lys	missense_variant	1	0.002557545	7.00E-06
DCM	TTN	c.107089G>C	p.Glu35697Gln	missense_variant	1	0.002557545	0.001663
DCM	TTN	c.106915G>C	p.Val35639Leu	missense_variant	1	0.002557545	0
DCM	TTN	c.106827T>G	p.Ile35609Met	missense_variant	1	0.002557545	0.001961
DCM	TTN	c.106787C>T	p.Thr35596Ile	missense_variant	1	0.002557545	0.007902
DCM	TTN	c.106675G>C	p.Glu35559Gln	missense_variant	6	0.015345269	0.000846
DCM	TTN	c.106619T>C	p.Ile35540Thr	missense_variant	6	0.015345269	0.016689
DCM	TTN	c.106580A>T	p.Glu35527Val	missense_variant	7	0.017902813	0.000794
DCM	TTN	c.106538A>G	p.Lys35513Arg	missense_variant	1	0.002557545	0
DCM	TTN	c.105788C>T	p.Ala35263Val	missense_variant	4	0.010230179	0.0157
DCM	TTN	c.105787G>T	p.Ala35263Ser	missense_variant	4	0.010230179	0.0157
DCM	TTN	c.105782C>T	p.Pro35261Le u	missense_variant	70	0.179028133	0.171254
DCM	TTN	c.105769G>A	p.Glu35257Lys	missense_variant	12	0.030690537	0.019658
DCM	TTN	c.105625A>C	p.Lys35209Gln	missense_variant	1	0.002557545	4.50E-05
DCM	TTN	c.105590G>A	p.Gly35197Asp	missense_variant	2	0.00511509	6.10E-05
DCM	TTN	c.105562A>C	p.Ile35188Leu	missense_variant	2	0.00511509	1.70E-05
DCM	TTN	c.105529G>A	p.Val35177Me t	missense_variant	15	0.038363171	0.033004
DCM	TTN	c.105180G>C	p.Glu35060As p	missense_variant	7	0.017902813	0.088113
DCM	TTN	c.104560G>C	p.Val34854Leu	missense_variant	1	0.002557545	0.007862
DCM	TTN	c.104377A>C	p.Met34793Le u	missense_variant	3	0.007672634	0.026232
DCM	TTN	c.104365G>A	p.Glu34789Lys	missense_variant	2	0.00511509	0.005022

DCM	TTN	c.104324G>T	p.Arg34775Leu	missense_variant	1	0.002557545	0
DCM	TTN	c.104251G>C	p.Ala34751Pro	missense_variant	1	0.002557545	0.000487
DCM	TTN	c.104125C>T	p.Arg34709Cys	missense_variant	1	0.002557545	2.20E-05
DCM	TTN	c.104027C>G	p.Thr34676Arg	missense_variant	2	0.00511509	0
DCM	TTN	c.103781G>A	p.Arg34594His	missense_variant	71	0.181585678	0.421851
DCM	TTN	c.103484T>C	p.Leu34495Pro	missense_variant	2	0.00511509	7.00E-06
DCM	TTN	c.103392G>C	p.Lys34464Asn	missense_variant	1	0.002557545	0
DCM	TTN	c.103363C>T	p.Arg34455Cys	missense_variant	4	0.010230179	0.008244
DCM	TTN	c.103074T>G	p.Asp34358Glu	missense_variant	1	0.002557545	0
DCM	TTN	c.102877A>G	p.Lys34293Glu	missense_variant	3	0.007672634	0.000314
DCM	TTN	c.102833G>T	p.Gly34278Val	missense_variant	65	0.166240409	0.170868
DCM	TTN	c.102757C>G	p.Leu34253Val	missense_variant	1	0.002557545	0
DCM	TTN	c.102713G>A	p.Arg34238His	missense_variant	1	0.002557545	4.40E-05
DCM	TTN	c.102595A>G	p.Ile34199Val	missense_variant	1	0.002557545	0.020366
DCM	TTN	c.102245C>A	p.Thr34082Asn	missense_variant	1	0.002557545	0
DCM	TTN	c.102194C>T	p.Ser34065Leu	missense_variant	1	0.002557545	0
DCM	TTN	c.102103G>A	p.Asp34035Asn	missense_variant	1	0.002557545	0.008484
DCM	TTN	c.101891G>A	p.Arg33964His	missense_variant	1	0.002557545	0.020807
DCM	TTN	c.101803A>G	p.Ile33935Val	missense_variant	5	0.012787724	0.049758
DCM	TTN	c.101766G>C	p.Gln33922His	missense_variant	7	0.017902813	0.010408
DCM	TTN	c.101669T>C	p.Met33890Thr	missense_variant	1	0.002557545	0
DCM	TTN	c.101291C>A	p.Ala33764Asp	missense_variant	1	0.002557545	0
DCM	TTN	c.101212C>T	p.Arg33738Cys	missense_variant	3	0.007672634	0.008204

DCM	TTN	c.100579G>A	p.Val33527Ile	missense_variant	62	0.158567775	0.151141
DCM	TTN	c.100459C>T	p.Pro33487Ser	missense_variant	3	0.007672634	0.037121
DCM	TTN	c.100449A>C	p.Glu33483Asp	missense_variant	3	0.007672634	2.60E-05
DCM	TTN	c.100400T>G	p.Val33467Gly	missense_variant	3	0.007672634	0.000441
DCM	TTN	c.100096G>A	p.Val33366Ile	missense_variant	1	0.002557545	0.063306
DCM	TTN	c.100094G>A	p.Arg33365Gln	missense_variant	2	0.00511509	0.019545
DCM	TTN	c.99991T>C	p.Cys33331Arg	missense_variant	9	0.023017903	0.000409
DCM	TTN	c.99922G>A	p.Ala33308Thr	missense_variant	1	0.002557545	2.90E-05
DCM	TTN	c.98912G>A	p.Arg32971His	missense_variant	17	0.043478261	0.190409
DCM	TTN	c.98862_98864delCAC	p.Thr32955del	inframe_deletion	1	0.002557545	0
DCM	TTN	c.98716G>A	p.Val32906Ile	missense_variant	1	0.002557545	0.000902
DCM	TTN	c.98659C>A	p.Pro32887Thr	missense_variant	1	0.002557545	0
DCM	TTN	c.98390A>G	p.Asn32797Ser	missense_variant	4	0.010230179	0.006708
DCM	TTN	c.98345G>T	p.Gly32782Val	missense_variant	1	0.002557545	0
DCM	TTN	c.98294C>G	p.Ala32765Gly	missense_variant	2	0.00511509	0.003958
DCM	TTN	c.98267C>T	p.Thr32756Ile	missense_variant	3	0.007672634	0.003727
DCM	TTN	c.98242C>T	p.Arg32748Cys	missense_variant	1	0.002557545	0.004902
DCM	TTN	c.98164A>T	p.Ile32722Phe	missense_variant	56	0.143222506	0.152116
DCM	TTN	c.97780G>A	p.Ala32594Thr	missense_variant	3	0.007672634	0
DCM	TTN	c.97769A>G	p.Lys32590Arg	missense_variant	1	0.002557545	0
DCM	TTN	c.97760G>A	p.Arg32587His	missense_variant	2	0.00511509	0.002562
DCM	TTN	c.97643G>A	p.Arg32548His	missense_variant	2	0.00511509	0.000169
DCM	TTN	c.97613G>A	p.Arg32538His	missense_variant	71	0.181585678	0.421379
DCM	TTN	c.97490T>C	p.Ile32497Thr	missense_variant,splice_region_variant	9	0.023017903	0.00717

DCM	TTN	c.97289C>A	p.Ala32430Asp	missense_variant	1	0.002557545	0
DCM	TTN	c.97099C>T	p.Arg32367Cys	missense_variant	4	0.010230179	0.001215
DCM	TTN	c.96944C>T	p.Thr32315Ile	missense_variant	4	0.010230179	0.062868
DCM	TTN	c.96521T>C	p.Met32174Th r	missense_variant	1	0.002557545	0.00011
DCM	TTN	c.96230G>A	p.Arg32077Gln	missense_variant	1	0.002557545	2.30E-05
DCM	TTN	c.96158T>C	p.Ile32053Thr	missense_variant	23	0.058823529	0.199103
DCM	TTN	c.96016G>A	p.Val32006Me t	missense_variant	3	0.007672634	0.000699
DCM	TTN	c.95999T>C	p.Ile32000Thr	missense_variant	2	0.00511509	3.00E-06
DCM	TTN	c.95582A>G	p.Tyr31861Cys	missense_variant	4	0.010230179	0.002631
DCM	TTN	c.95555T>C	p.Leu31852Pr o	missense_variant	6	0.015345269	0.016728
DCM	TTN	c.95415C>A	p.Phe31805Le u	missense_variant ,splice_region_va riant	9	0.023017903	4.80E-05
DCM	TTN	c.95297C>T	p.Ser31766Ph e	missense_variant	1	0.002557545	0.020331
DCM	TTN	c.95242C>T	p.Arg31748Cys	missense_variant	8	0.020460358	0.000908
DCM	TTN	c.95047A>G	p.Ser31683Gly	missense_variant	1	0.002557545	0.058704
DCM	TTN	c.94851T>A	p.Asp31617Gl u	missense_variant	15	0.038363171	0.0051
DCM	TTN	c.94840G>A	p.Ala31614Thr	missense_variant	1	0.002557545	0
DCM	TTN	c.94774G>A	p.Val31592Ile	missense_variant	1	0.002557545	2.30E-05
DCM	TTN	c.94282C>G	p.Arg31428Gly	missense_variant	1	0.002557545	0.000699
DCM	TTN	c.94211A>G	p.His31404Arg	missense_variant	1	0.002557545	0
DCM	TTN	c.94046G>A	p.Arg31349His	missense_variant	1	0.002557545	0.001838
DCM	TTN	c.93901G>A	p.Val31301Ile	missense_variant	15	0.038363171	0.109907
DCM	TTN	c.93430C>T	p.Arg31144Trp	missense_variant	2	0.00511509	0

DCM	TTN	c.93316G>C	p.Glu31106Gln	missense_variant	1	0.002557545	0
DCM	TTN	c.93215G>A	p.Arg31072His	missense_variant	1	0.002557545	7.00E-04
DCM	TTN	c.92905C>T	p.Arg30969Trp	missense_variant	1	0.002557545	3.00E-06
DCM	TTN	c.92780T>C	p.Ile30927Thr	missense_variant	1	0.002557545	0
DCM	TTN	c.92537T>C	p.Val30846Ala	missense_variant	1	0.002557545	0.008304
DCM	TTN	c.92504A>G	p.Lys30835Arg	missense_variant	1	0.002557545	0
DCM	TTN	c.92311A>G	p.Ser30771Gly	missense_variant	1	0.002557545	0
DCM	TTN	c.92191A>G	p.Ile30731Val	missense_variant	2	0.00511509	0.220218
DCM	TTN	c.92176C>T	p.Pro30726Ser	missense_variant	1	0.002557545	0.004605
DCM	TTN	c.92131G>A	p.Val30711Met	missense_variant	37	0.094629156	0.252258
DCM	TTN	c.91937A>G	p.Asn30646Ser	missense_variant	1	0.002557545	0.004962
DCM	TTN	c.91868T>C	p.Val30623Ala	missense_variant	1	0.002557545	0
DCM	TTN	c.91765G>A	p.Ala30589Thr	missense_variant	1	0.002557545	0.020337
DCM	TTN	c.91601A>T	p.Asp30534Val	missense_variant	2	0.00511509	0.000872
DCM	TTN	c.91589C>T	p.Pro30530Leu	missense_variant	1	0.002557545	0.003805
DCM	TTN	c.91306C>T	p.Arg30436Trp	missense_variant	1	0.002557545	1.00E-05
DCM	TTN	c.91192C>T	p.Arg30398Cys	missense_variant	1	0.002557545	3.00E-06
DCM	TTN	c.90968G>A	p.Arg30323Lys	missense_variant	15	0.038363171	0.109766
DCM	TTN	c.90826T>G	p.Cys30276Gly	missense_variant	1	0.002557545	0.019537
DCM	TTN	c.90638T>C	p.Ile30213Thr	missense_variant	1	0.002557545	0.019921
DCM	TTN	c.90589G>A	p.Glu30197Lys	missense_variant	1	0.002557545	0
DCM	TTN	c.90536G>A	p.Arg30179His	missense_variant	1	0.002557545	0.020674
DCM	TTN	c.89386G>A	p.Val29796Met	missense_variant	2	0.00511509	0.004782
DCM	TTN	c.89317A>T	p.Ile29773Leu	missense_variant	15	0.038363171	0.032999
DCM	TTN	c.89018G>A	p.Arg29673Gln	missense_variant	3	0.007672634	0.000576

DCM	TTN	c.88946C>T	p.Ser29649Leu	missense_variant	2	0.00511509	9.70E-05
DCM	TTN	c.88708A>G	p.Ile29570Val	missense_variant	9	0.023017903	0.007281
DCM	TTN	c.88510G>T	p.Asp29504Tyr	missense_variant	1	0.002557545	0
DCM	TTN	c.88187T>C	p.Ile29396Thr	missense_variant	188	0.480818414	0.693579
DCM	TTN	c.88090G>A	p.Gly29364Ser	missense_variant	1	0.002557545	0.000537
DCM	TTN	c.87877C>T	p.Arg29293Cys	missense_variant	1	0.002557545	0.02406
DCM	TTN	c.87808G>A	p.Val29270Ile	missense_variant	1	0.002557545	0.00414
DCM	TTN	c.87448A>T	p.Ile29150Leu	missense_variant	1	0.002557545	2.30E-05
DCM	TTN	c.87412C>A	p.Pro29138Thr	missense_variant	4	0.010230179	0.00314
DCM	TTN	c.86471C>T	p.Thr28824Ile	missense_variant	1	0.002557545	0.000115
DCM	TTN	c.85553G>A	p.Gly28518Asp	missense_variant	1	0.002557545	0
DCM	TTN	c.85350G>C	p.Lys28450Asn	missense_variant	1	0.002557545	3.00E-06
DCM	TTN	c.85316G>A	p.Arg28439Gln	missense_variant	1	0.002557545	1.00E-05
DCM	TTN	c.85265G>A	p.Arg28422Lys	missense_variant	1	0.002557545	0
DCM	TTN	c.84976C>T	p.Arg28326Trp	missense_variant	1	0.002557545	1.70E-05
DCM	TTN	c.84923A>C	p.Gln28308Pro	missense_variant	1	0.002557545	0.000276
DCM	TTN	c.84883C>T	p.Pro28295Ser	missense_variant	1	0.002557545	0
DCM	TTN	c.84352C>T	p.Arg28118Cys	missense_variant	4	0.010230179	0.062819
DCM	TTN	c.83740A>G	p.Thr27914Ala	missense_variant	9	0.023017903	0.007239
DCM	TTN	c.83608G>A	p.Val27870Ile	missense_variant	1	0.002557545	0
DCM	TTN	c.83516G>A	p.Arg27839Gln	missense_variant	4	0.010230179	0.000744
DCM	TTN	c.83323A>G	p.Ile27775Val	missense_variant	189	0.483375959	0.693349
DCM	TTN	c.83299C>A	p.Pro27767Thr	missense_variant	1	0.002557545	0.00175
DCM	TTN	c.83171T>G	p.Val27724Gly	missense_variant	5	0.012787724	0.000132
DCM	TTN	c.83056G>A	p.Val27686Ile	missense_variant	6	0.015345269	0.062841
DCM	TTN	c.82798G>A	p.Ala27600Thr	missense_variant	15	0.038363171	0.108845
DCM	TTN	c.82560C>A	p.Asn27520Lys	missense_variant	1	0.002557545	0.019635

DCM	TTN	c.82385C>A	p.Thr27462Lys	missense_variant	25	0.063938619	0.047588
DCM	TTN	c.81958G>A	p.Ala27320Thr	missense_variant	4	0.010230179	0.062811
DCM	TTN	c.81250A>G	p.Ile27084Val	missense_variant	1	0.002557545	0.000127
DCM	TTN	c.81035A>G	p.Lys27012Arg	missense_variant	1	0.002557545	0
DCM	TTN	c.80822_80824delAAG	p.Glu26941del	inframe_deletion	1	0.002557545	0
DCM	TTN	c.80701A>G	p.Ile26901Val	missense_variant	7	0.017902813	0.000393
DCM	TTN	c.80635C>A	p.Gln26879Lys	missense_variant	5	0.012787724	0.046409
DCM	TTN	c.80426G>A	p.Gly26809Asp	missense_variant	1	0.002557545	1.90E-05
DCM	TTN	c.79862C>T	p.Thr26621Met	missense_variant	160	0.409207161	0.603621
DCM	TTN	c.79783G>C	p.Asp26595His	missense_variant	13	0.033248082	0.019119
DCM	TTN	c.79685G>A	p.Arg26562Gln	missense_variant	1	0.002557545	0.000226
DCM	TTN	c.79319G>A	p.Arg26440His	missense_variant	2	0.00511509	0.013178
DCM	TTN	c.79318C>T	p.Arg26440Cys	missense_variant	2	0.00511509	0.024974
DCM	TTN	c.79265T>C	p.Ile26422Thr	missense_variant	1	0.002557545	0.058746
DCM	TTN	c.79178T>C	p.Leu26393Ser	missense_variant	3	0.007672634	0
DCM	TTN	c.78674T>C	p.Ile26225Thr	missense_variant	122	0.31202046	0.302485
DCM	TTN	c.78304A>G	p.Arg26102Gly	missense_variant	1	0.002557545	0.000177
DCM	TTN	c.78019A>G	p.Met26007Val	missense_variant	1	0.002557545	0
DCM	TTN	c.77975G>A	p.Gly25992Asp	missense_variant	1	0.002557545	0
DCM	TTN	c.77638A>G	p.Thr25880Ala	missense_variant	1	0.002557545	0.058713
DCM	TTN	c.77522C>T	p.Thr25841Ile	missense_variant	1	0.002557545	0
DCM	TTN	c.77279A>G	p.Asn25760Ser	missense_variant	1	0.002557545	0.058792
DCM	TTN	c.76838T>A	p.Ile25613Asn	missense_variant	1	0.002557545	0
DCM	TTN	c.76720T>C	p.Tyr25574His	missense_variant	1	0.002557545	0.058895
DCM	TTN	c.76483G>A	p.Val25495Ile	missense_variant	1	0.002557545	0.000908

DCM	TTN	c.76343G>A	p.Ser25448Asn	missense_variant	70	0.179028133	0.175649
DCM	TTN	c.75734G>A	p.Arg25245Lys	missense_variant	1	0.002557545	1.70E-05
DCM	TTN	c.75483G>C	p.Lys25161Asn	missense_variant	1	0.002557545	0
DCM	TTN	c.75262G>C	p.Gly25088Arg	missense_variant	2	0.00511509	0
DCM	TTN	c.75065C>T	p.Ser25022Phe	missense_variant	1	0.002557545	0
DCM	TTN	c.74972T>C	p.Ile24991Thr	missense_variant	5	0.012787724	0.048335
DCM	TTN	c.74839C>T	p.Arg24947Cys	missense_variant	71	0.181585678	0.420168
DCM	TTN	c.74774T>C	p.Met24925Thr	missense_variant	1	0.002557545	0
DCM	TTN	c.74377T>A	p.Ser24793Thr	missense_variant	1	0.002557545	0
DCM	TTN	c.73825G>C	p.Glu24609Gln	missense_variant	2	0.00511509	0.003433
DCM	TTN	c.73766A>G	p.Tyr24589Cys	missense_variant	1	0.002557545	0
DCM	TTN	c.73435A>G	p.Ser24479Gly	missense_variant	1	0.002557545	0
DCM	TTN	c.73304G>T	p.Arg24435Leu	missense_variant	2	0.00511509	0
DCM	TTN	c.73263C>G	p.Asp24421Glu	missense_variant	1	0.002557545	0
DCM	TTN	c.73102G>A	p.Asp24368Asn	missense_variant	1	0.002557545	0
DCM	TTN	c.72766A>G	p.Asn24256Asp	missense_variant	1	0.002557545	0.000847
DCM	TTN	c.72664C>T	p.Pro24222Ser	missense_variant	1	0.002557545	0
DCM	TTN	c.72598G>C	p.Val24200Leu	missense_variant	1	0.002557545	0
DCM	TTN	c.71993G>A	p.Arg23998His	missense_variant	19	0.04859335	0.196244
DCM	TTN	c.71841G>C	p.Lys23947Asn	missense_variant	1	0.002557545	0.001231
DCM	TTN	c.71354C>T	p.Thr23785Ile	missense_variant	1	0.002557545	0
DCM	TTN	c.70817T>C	p.Met23606Thr	missense_variant	1	0.002557545	0.000257

DCM	TTN	c.70753G>A	p.Val23585Met	missense_variant	2	0.00511509	1.00E-05
DCM	TTN	c.70700A>C	p.Tyr23567Ser	missense_variant	1	0.002557545	0
DCM	TTN	c.70640G>C	p.Ser23547Thr	missense_variant	1	0.002557545	0
DCM	TTN	c.70417G>A	p.Glu23473Lys	missense_variant	1	0.002557545	0
DCM	TTN	c.70172T>C	p.Ile23391Thr	missense_variant	1	0.002557545	1.00E-05
DCM	TTN	c.70102A>G	p.Ile23368Val	missense_variant	2	0.00511509	0.000103
DCM	TTN	c.70015G>C	p.Glu23339Gln	missense_variant	1	0.002557545	0
DCM	TTN	c.69903C>A	p.Phe23301Leu	missense_variant	1	0.002557545	0.001673
DCM	TTN	c.69773C>A	p.Ser23258Tyr	missense_variant	1	0.002557545	0
DCM	TTN	c.69676A>G	p.Ser23226Gly	missense_variant	8	0.020460358	0.010332
DCM	TTN	c.69646G>A	p.Ala23216Thr	missense_variant	3	0.007672634	0
DCM	TTN	c.69145A>G	p.Ile23049Val	missense_variant	16	0.040920716	0.111007
DCM	TTN	c.69130C>T	p.Pro23044Ser	missense_variant	1	0.002557545	0.005135
DCM	TTN	c.69044C>T	p.Ala23015Val	missense_variant	1	0.002557545	4.80E-05
DCM	TTN	c.68824G>A	p.Glu22942Lys	missense_variant,splice_region_variant	1	0.002557545	0.000331
DCM	TTN	c.68458G>C	p.Ala22820Pro	missense_variant	2	0.00511509	0.002302
DCM	TTN	c.68215C>T	p.His22739Tyr	missense_variant	1	0.002557545	0
DCM	TTN	c.67605C>G	p.Ser22535Arg	missense_variant	1	0.002557545	3.00E-06
DCM	TTN	c.67445G>A	p.Arg22482Gln	missense_variant	1	0.002557545	0.000157
DCM	TTN	c.67246G>C	p.Ala22416Pro	missense_variant	391	1	0.994762
DCM	TTN	c.67075G>A	p.Val22359Ile	missense_variant	163	0.416879795	0.600982
DCM	TTN	c.66977A>G	p.Lys22326Arg	missense_variant	1	0.002557545	0.004257
DCM	TTN	c.66658A>G	p.Thr22220Ala	missense_variant	1	0.002557545	0
DCM	TTN	c.66614G>A	p.Arg22205Lys	missense_variant	4	0.010230179	0.02139

DCM	TTN	c.66376T>C	p.Tyr22126His	missense_variant	1	0.002557545	0
DCM	TTN	c.66306G>T	p.Trp22102Cys	missense_variant	1	0.002557545	0
DCM	TTN	c.66086G>A	p.Arg22029His	missense_variant	1	0.002557545	4.80E-05
DCM	TTN	c.65956T>C	p.Ser21986Pro	missense_variant	1	0.002557545	0
DCM	TTN	c.65743C>A	p.Gln21915Lys	missense_variant	5	0.012787724	0.047294
DCM	TTN	c.65737G>T	p.Ala21913Ser	missense_variant	2	0.00511509	0
DCM	TTN	c.65534C>T	p.Pro21845Leu	missense_variant	2	0.00511509	0.000158
DCM	TTN	c.65516C>T	p.Ala21839Val	missense_variant	9	0.023017903	0.04151
DCM	TTN	c.65503A>G	p.Ile21835Val	missense_variant	1	0.002557545	0
DCM	TTN	c.65459C>T	p.Thr21820Ile	missense_variant	9	0.023017903	0.00097
DCM	TTN	c.65147C>T	p.Ser21716Leu	missense_variant	1	0.002557545	0.038886
DCM	TTN	c.65092C>T	p.Arg21698Cys	missense_variant	9	0.023017903	0.034094
DCM	TTN	c.64789G>A	p.Val21597Met	missense_variant	1	0.002557545	0.020333
DCM	TTN	c.64787T>C	p.Ile21596Thr	missense_variant	1	0.002557545	0
DCM	TTN	c.64762G>A	p.Gly21588Arg	missense_variant	9	0.023017903	0.006993
DCM	TTN	c.64654A>G	p.Ile21552Val	missense_variant	1	0.002557545	0.001078
DCM	TTN	c.64283T>C	p.Val21428Ala	missense_variant	1	0.002557545	0
DCM	TTN	c.64208C>T	p.Thr21403Ile	missense_variant	187	0.47826087	0.69818
DCM	TTN	c.63578G>A	p.Arg21193His	missense_variant	1	0.002557545	4.40E-05
DCM	TTN	c.63463C>T	p.Arg21155Cys	missense_variant	1	0.002557545	1.10E-05
DCM	TTN	c.63109C>T	p.Arg21037Cys	missense_variant	1	0.002557545	0.000359
DCM	TTN	c.63026G>A	p.Arg21009Gln	missense_variant	2	0.00511509	0.021906
DCM	TTN	c.63023C>T	p.Thr21008Ile	missense_variant	11	0.028132992	0.01356
DCM	TTN	c.62567A>G	p.Tyr20856Cys	missense_variant	1	0.002557545	0.000168
DCM	TTN	c.62237A>C	p.Lys20746Thr	missense_variant	1	0.002557545	0

DCM	TTN	c.62137G>A	p.Asp20713As n	missense_variant	1	0.002557545	0
DCM	TTN	c.61922G>A	p.Arg20641Gln	missense_variant	2	0.00511509	0.003882
DCM	TTN	c.61853T>C	p.Ile20618Thr	missense_variant	1	0.002557545	0
DCM	TTN	c.61556G>A	p.Arg20519Gln	missense_variant	1	0.002557545	0.000179
DCM	TTN	c.61073C>T	p.Pro20358Le u	missense_variant	1	0.002557545	0
DCM	TTN	c.60821C>T	p.Pro20274Le u	missense_variant	5	0.012787724	0.016253
DCM	TTN	c.60524C>T	p.Pro20175Le u	missense_variant	2	0.00511509	5.40E-05
DCM	TTN	c.60490G>C	p.Val20164Leu	missense_variant	2	0.00511509	0.012495
DCM	TTN	c.60314T>G	p.Val20105Gly	missense_variant	2	0.00511509	1.20E-05
DCM	TTN	c.60242A>G	p.Asp20081Gly	missense_variant	1	0.002557545	0
DCM	TTN	c.60232G>A	p.Val20078Me t	missense_variant	2	0.00511509	0.022165
DCM	TTN	c.59812G>A	p.Ala19938Thr	missense_variant	1	0.002557545	0
DCM	TTN	c.59729C>T	p.Thr19910Ile	missense_variant	2	0.00511509	4.80E-05
DCM	TTN	c.59700A>C	p.Glu19900As p	missense_variant	1	0.002557545	0
DCM	TTN	c.59585C>T	p.Pro19862Le u	missense_variant	71	0.181585678	0.42401
DCM	TTN	c.59563T>A	p.Tyr19855Asn	missense_variant	1	0.002557545	0
DCM	TTN	c.59165T>C	p.Val19722Ala	missense_variant	4	0.010230179	0.011695
DCM	TTN	c.58930C>A	p.Leu19644Ile	missense_variant	1	0.002557545	0
DCM	TTN	c.58636G>C	p.Glu19546Gln	missense_variant	2	0.00511509	0.009094
DCM	TTN	c.58436G>A	p.Arg19479His	missense_variant	71	0.181585678	0.422752
DCM	TTN	c.58363G>A	p.Gly19455Ser	missense_variant	1	0.002557545	0.000207
DCM	TTN	c.58288G>A	p.Asp19430As n	missense_variant	1	0.002557545	0

DCM	TTN	c.58202G>C	p.Gly19401Ala	missense_variant	1	0.002557545	0
DCM	TTN	c.57899C>A	p.Thr19300Asn	missense_variant	1	0.002557545	0
DCM	TTN	c.57860G>A	p.Arg19287His	missense_variant	2	0.00511509	0.000119
DCM	TTN	c.57464G>A	p.Arg19155Lys	missense_variant	3	0.007672634	0.019491
DCM	TTN	c.57370G>A	p.Val19124Ile	missense_variant	1	0.002557545	0.000962
DCM	TTN	c.57052T>G	p.Ser19018Ala	missense_variant	1	0.002557545	3.00E-06
DCM	TTN	c.56787G>C	p.Lys18929Asn	missense_variant	1	0.002557545	0
DCM	TTN	c.56702T>C	p.Met18901Thr	missense_variant	1	0.002557545	0
DCM	TTN	c.56632G>A	p.Ala18878Thr	missense_variant	2	0.00511509	0
DCM	TTN	c.56591A>C	p.Lys18864Thr	missense_variant	1	0.002557545	0
DCM	TTN	c.56422C>T	p.Pro18808Ser	missense_variant	3	0.007672634	2.30E-05
DCM	TTN	c.56101A>G	p.Asn18701Asp	missense_variant	194	0.496163683	0.702145
DCM	TTN	c.55553A>G	p.Lys18518Arg	missense_variant	17	0.043478261	0.005321
DCM	TTN	c.55139T>C	p.Ile18380Thr	missense_variant	2	0.00511509	9.90E-05
DCM	TTN	c.54748A>C	p.Asn18250His	missense_variant	1	0.002557545	0
DCM	TTN	c.54741G>A	p.Met18247Ile	missense_variant	1	0.002557545	3.00E-06
DCM	TTN	c.54511G>A	p.Gly18171Arg	missense_variant	2	0.00511509	0
DCM	TTN	c.54419G>A	p.Arg18140Gln	missense_variant	2	0.00511509	0
DCM	TTN	c.53903G>A	p.Arg17968His	missense_variant	1	0.002557545	9.30E-05
DCM	TTN	c.53192T>C	p.Ile17731Thr	missense_variant	9	0.023017903	0.013427
DCM	TTN	c.53167G>A	p.Val17723Ile	missense_variant	1	0.002557545	0
DCM	TTN	c.53123A>T	p.Lys17708Ile	missense_variant	1	0.002557545	0.098717
DCM	TTN	c.52144A>G	p.Arg17382Gly	missense_variant	1	0.002557545	0.001916
DCM	TTN	c.51896C>T	p.Pro17299Leu	missense_variant	2	0.00511509	8.20E-05
DCM	TTN	c.51887G>A	p.Arg17296His	missense_variant	1	0.002557545	0.000554

DCM	TTN	c.51885G>T	p.Lys17295Asn	missense_variant	1	0.002557545	0
DCM	TTN	c.51737T>C	p.Ile17246Thr	missense_variant,splice_region_variant	1	0.002557545	0
DCM	TTN	c.51579G>C	p.Arg17193Ser	missense_variant	1	0.002557545	1.20E-05
DCM	TTN	c.51482C>T	p.Ala17161Val	missense_variant	24	0.061381074	0.127692
DCM	TTN	c.50155T>C	p.Ser16719Pro	missense_variant	2	0.00511509	0
DCM	TTN	c.50045C>T	p.Pro16682Leu	missense_variant	1	0.002557545	0
DCM	TTN	c.49985A>C	p.Asn16662Thr	missense_variant	20	0.051150895	0.020799
DCM	TTN	c.49919G>C	p.Ser16640Thr	missense_variant	1	0.002557545	0.020385
DCM	TTN	c.49691G>T	p.Gly16564Val	missense_variant	1	0.002557545	0
DCM	TTN	c.49265T>C	p.Ile16422Thr	missense_variant	2	0.00511509	0
DCM	TTN	c.48953T>C	p.Ile16318Thr	missense_variant	5	0.012787724	0.001055
DCM	TTN	c.48838G>A	p.Ala16280Thr	missense_variant	1	0.002557545	9.50E-05
DCM	TTN	c.48751G>A	p.Asp16251Asn	missense_variant	1	0.002557545	1.70E-05
DCM	TTN	c.48725G>T	p.Arg16242Ile	missense_variant	1	0.002557545	0
DCM	TTN	c.48164G>A	p.Arg16055His	missense_variant	1	0.002557545	0.00017
DCM	TTN	c.48064A>C	p.Ile16022Leu	missense_variant	1	0.002557545	0
DCM	TTN	c.47723G>A	p.Arg15908His	missense_variant	7	0.017902813	0.009746
DCM	TTN	c.47693G>A	p.Arg15898Gln	missense_variant	1	0.002557545	9.00E-05
DCM	TTN	c.47545C>A	p.Pro15849Thr	missense_variant	1	0.002557545	0.020404
DCM	TTN	c.47315G>A	p.Arg15772Gln	missense_variant	18	0.046035806	0.002867
DCM	TTN	c.47191C>T	p.Arg15731Cys	missense_variant	3	0.007672634	0.003313
DCM	TTN	c.47129G>A	p.Arg15710His	missense_variant	1	0.002557545	0.000146
DCM	TTN	c.46880C>T	p.Ala15627Val	missense_variant	2	0.00511509	0.005032
DCM	TTN	c.46823T>C	p.Leu15608Ser	missense_variant	1	0.002557545	0.001101

DCM	TTN	c.46693G>T	p.Ala15565Ser	missense_variant	1	0.002557545	0.004865
DCM	TTN	c.46521G>C	p.Lys15507Asn	missense_variant	1	0.002557545	0
DCM	TTN	c.45980G>A	p.Arg15327His	missense_variant	1	0.002557545	4.60E-05
DCM	TTN	c.45601C>G	p.His15201Asp	missense_variant	1	0.002557545	0
DCM	TTN	c.45499G>A	p.Val15167Ile	missense_variant	1	0.002557545	0.001843
DCM	TTN	c.45408G>T	p.Lys15136Asn	missense_variant	1	0.002557545	0.007362
DCM	TTN	c.45283G>A	p.Glu15095Lys	missense_variant	1	0.002557545	0
DCM	TTN	c.45206A>T	p.Glu15069Val	missense_variant	31	0.079283887	0.053263
DCM	TTN	c.45175G>A	p.Ala15059Thr	missense_variant	1	0.002557545	0.01403
DCM	TTN	c.44423A>C	p.Lys14808Thr	missense_variant,splice_region_variant	3	0.007672634	9.50E-05
DCM	TTN	c.44350G>A	p.Asp14784Asn	missense_variant	1	0.002557545	2.60E-05
DCM	TTN	c.44222C>T	p.Thr14741Met	missense_variant	1	0.002557545	2.60E-05
DCM	TTN	c.44062G>C	p.Glu14688Gln	missense_variant	1	0.002557545	0
DCM	TTN	c.43502C>G	p.Thr14501Ser	missense_variant	7	0.017902813	0.000479
DCM	TTN	c.43138T>C	p.Cys14380Arg	missense_variant	1	0.002557545	0.002781
DCM	TTN	c.43045G>A	p.Gly14349Ser	missense_variant	1	0.002557545	3.90E-05
DCM	TTN	c.42958A>G	p.Lys14320Glu	missense_variant	42	0.10741688	0.105863
DCM	TTN	c.42862A>G	p.Ile14288Val	missense_variant	1	0.002557545	0
DCM	TTN	c.42333T>G	p.Ile14111Met	missense_variant	1	0.002557545	0
DCM	TTN	c.42071A>G	p.His14024Arg	missense_variant	1	0.002557545	0.09893
DCM	TTN	c.41158A>G	p.Met13720Val	missense_variant	1	0.002557545	0
DCM	TTN	c.41088A>C	p.Glu13696Asp	missense_variant	1	0.002557545	0
DCM	TTN	c.40973A>G	p.Lys13658Arg	missense_variant	1	0.002557545	0.000155

DCM	TTN	c.40831C>G	p.Pro13611Ala	missense_variant	1	0.002557545	0
DCM	TTN	c.40585_40587dupGAA	p.Glu13529dup	inframe_insertion	3	0.007672634	0
DCM	TTN	c.40543G>A	p.Val13515Ile	missense_variant	1	0.002557545	0.000196
DCM	TTN	c.40349C>T	p.Pro13450Leu	missense_variant	1	0.002557545	0
DCM	TTN	c.40223A>G	p.Glu13408Gly	missense_variant,splice_region_variant	3	0.007672634	0.002335
DCM	TTN	c.39749_39766delTTGCTCCTGAAGAGGAAA	p.Ile13250_Glu13255del	inframe_deletion	16	0.040920716	0.042984
DCM	TTN	c.39704C>G	p.Pro13235Arg	missense_variant	9	0.023017903	0.117208
DCM	TTN	c.39689C>T	p.Ala13230Val	missense_variant	1	0.002557545	0.001136
DCM	TTN	c.39673C>G	p.Pro13225Ala	missense_variant	1	0.002557545	0
DCM	TTN	c.39082G>A	p.Val13028Met	missense_variant	4	0.010230179	0.03071
DCM	TTN	c.38902C>T	p.Pro12968Ser	missense_variant	2	0.00511509	0.003047
DCM	TTN	c.38803C>T	p.Pro12935Ser	missense_variant	1	0.002557545	0
DCM	TTN	c.38755G>A	p.Ala12919Thr	missense_variant	3	0.007672634	0.000354
DCM	TTN	c.38753T>C	p.Leu12918Ser	missense_variant	21	0.05370844	0.219614
DCM	TTN	c.38386G>A	p.Glu12796Lys	missense_variant	2	0.00511509	0
DCM	TTN	c.38336T>C	p.Val12779Ala	missense_variant	7	0.017902813	0.003937
DCM	TTN	c.38311A>G	p.Lys12771Glu	missense_variant	6	0.015345269	0.001922
DCM	TTN	c.37247C>T	p.Ser12416Leu	missense_variant	2	0.00511509	0.001202
DCM	TTN	c.37009C>T	p.Pro12337Ser	missense_variant	5	0.012787724	0.025209
DCM	TTN	c.36625G>T	p.Val12209Leu	missense_variant	16	0.040920716	0.156869
DCM	TTN	c.36590A>C	p.Lys12197Thr	missense_variant	1	0.002557545	0

DCM	TTN	c.36508G>A	p.Glu12170Lys	missense_variant	84	0.21483376	0.462977
DCM	TTN	c.36461C>G	p.Pro12154Arg	missense_variant	1	0.002557545	0.000454
DCM	TTN	c.36299A>T	p.Glu12100Val	missense_variant	24	0.061381074	0.089634
DCM	TTN	c.36196G>A	p.Asp12066Asn	missense_variant	1	0.002557545	0.00038
DCM	TTN	c.36126A>C	p.Glu12042Asp	missense_variant	1	0.002557545	0.019684
DCM	TTN	c.35335G>A	p.Val11779Ile	missense_variant	1	0.002557545	0
DCM	TTN	c.34970G>A	p.Arg11657His	missense_variant	2	0.00511509	0.024307
DCM	TTN	c.34571G>A	p.Arg11524Gln	missense_variant	1	0.002557545	0.000527
DCM	TTN	c.34566A>C	p.Glu11522Asp	missense_variant	1	0.002557545	0.019242
DCM	TTN	c.34474C>A	p.Pro11492Thr	missense_variant	6	0.015345269	0.005035
DCM	TTN	c.34345C>T	p.Pro11449Ser	missense_variant	1	0.002557545	0
DCM	TTN	c.34247_34249delAAG	p.Glu11416del	inframe_deletion	2	0.00511509	0.01713
DCM	TTN	c.34072G>T	p.Val11358Phe	missense_variant	1	0.002557545	0
DCM	TTN	c.33862_33864delAAG	p.Lys11288del	inframe_deletion	1	0.002557545	3.00E-05
DCM	TTN	c.33754C>A	p.Pro11252Thr	missense_variant	1	0.002557545	0
DCM	TTN	c.33479C>T	p.Thr11160Ile	missense_variant	2	0.00511509	0
DCM	TTN	c.33287G>A	p.Arg11096His	missense_variant	127	0.324808184	0.30036
DCM	TTN	c.33127C>T	p.Pro11043Ser	missense_variant	1	0.002557545	3.00E-06
DCM	TTN	c.33053G>A	p.Arg11018Gln	missense_variant	3	0.007672634	0.000844
DCM	TTN	c.32624C>T	p.Pro10875Leu	missense_variant	15	0.038363171	0.006111
DCM	TTN	c.32593G>C	p.Val10865Leu	missense_variant	3	0.007672634	0.003439

DCM	TTN	c.32557C>T	p.Pro10853Ser	missense_variant ,splice_region_variant	4	0.010230179	0.000952
DCM	TTN	c.32512C>G	p.Pro10838Ala	missense_variant	2	0.00511509	0
DCM	TTN	c.32482C>T	p.Pro10828Ser	missense_variant	1	0.002557545	1.00E-05
DCM	TTN	c.32350C>G	p.Leu10784Val	missense_variant	23	0.058823529	0.010444
DCM	TTN	c.32254G>A	p.Val10752Ile	missense_variant	2	0.00511509	0.036434
DCM	TTN	c.31884A>T	p.Lys10628Asn	missense_variant	1	0.002557545	3.00E-06
DCM	TTN	c.31864G>A	p.Gly10622Arg	missense_variant	207	0.529411765	0.59641
DCM	TTN	c.31837C>G	p.Pro10613Ala	missense_variant	13	0.033248082	0.000533
DCM	TTN	c.31564A>G	p.Ile10522Val	missense_variant	196	0.501278772	0.564398
DCM	TTN	c.31399G>A	p.Val10467Ile	missense_variant	1	0.002557545	0.002999
DCM	TTN	c.31390C>T	p.Arg10464Trp	missense_variant	1	0.002557545	3.50E-05
DCM	TTN	c.30952G>A	p.Glu10318Lys	missense_variant	16	0.040920716	0.110899
DCM	TTN	c.30629C>G	p.Pro10210Arg	missense_variant	2	0.00511509	1.20E-05
DCM	TTN	c.30274C>T	p.His10092Tyr	missense_variant	1	0.002557545	0.005053
DCM	TTN	c.29812A>T	p.Thr9938Ser	missense_variant	15	0.038363171	0.024652
DCM	TTN	c.29153T>C	p.Ile9718Thr	missense_variant	61	0.15601023	0.197919
DCM	TTN	c.29119A>G	p.Ile9707Val	missense_variant	1	0.002557545	0
DCM	TTN	c.28765C>T	p.Pro9589Ser	missense_variant	1	0.002557545	0
DCM	TTN	c.28507G>A	p.Val9503Ile	missense_variant	1	0.002557545	0.000479
DCM	TTN	c.28354G>A	p.Ala9452Thr	missense_variant	1	0.002557545	0.000226
DCM	TTN	c.28313G>A	p.Arg9438Gln	missense_variant	8	0.020460358	0.092033
DCM	TTN	c.28070C>T	p.Thr9357Ile	missense_variant	1	0.002557545	0.004489
DCM	TTN	c.27799G>T	p.Val9267Phe	missense_variant	1	0.002557545	0
DCM	TTN	c.27677G>A	p.Cys9226Tyr	missense_variant	2	0.00511509	3.00E-06
DCM	TTN	c.26818G>A	p.Gly8940Ser	missense_variant	3	0.007672634	0.002226
DCM	TTN	c.26681C>T	p.Pro8894Leu	missense_variant	42	0.10741688	0.186432

DCM	TTN	c.26672A>G	p.Asn8891Ser	missense_variant	11	0.028132992	0.00048
DCM	TTN	c.26641T>G	p.Phe8881Val	missense_variant	1	0.002557545	0
DCM	TTN	c.26597A>G	p.Asp8866Gly	missense_variant	1	0.002557545	0
DCM	TTN	c.26468C>T	p.Thr8823Met	missense_variant	1	0.002557545	2.20E-05
DCM	TTN	c.26408A>G	p.Asn8803Ser	missense_variant	122	0.31202046	0.422846
DCM	TTN	c.26245G>A	p.Val8749Ile	missense_variant	12	0.030690537	0.039189
DCM	TTN	c.26056G>A	p.Gly8686Ser	missense_variant	1	0.002557545	0.000313
DCM	TTN	c.25978G>A	p.Val8660Ile	missense_variant	6	0.015345269	0.009878
DCM	TTN	c.25936C>T	p.Arg8646Cys	missense_variant	13	0.033248082	0.020396
DCM	TTN	c.25660A>G	p.Lys8554Glu	missense_variant	1	0.002557545	0.000125
DCM	TTN	c.25626G>T	p.Gln8542His	missense_variant	1	0.002557545	0.204574
DCM	TTN	c.25490G>A	p.Arg8497His	missense_variant	1	0.002557545	0.020489
DCM	TTN	c.25453A>G	p.Ile8485Val	missense_variant	1	0.002557545	0
DCM	TTN	c.25398T>A	p.Asp8466Glu	missense_variant	14	0.035805627	0.071651
DCM	TTN	c.25274G>A	p.Ser8425Asn	missense_variant	117	0.299232737	0.421681
DCM	TTN	c.25087G>T	p.Ala8363Ser	missense_variant	1	0.002557545	0.001932
DCM	TTN	c.25064C>A	p.Ala8355Glu	missense_variant ,splice_region_variant	171	0.437340153	0.657331
DCM	TTN	c.24880A>G	p.Arg8294Gly	missense_variant	33	0.084398977	0.029338
DCM	TTN	c.24652A>G	p.Ser8218Gly	missense_variant	1	0.002557545	0.007042
DCM	TTN	c.24431A>C	p.Glu8144Ala	missense_variant	115	0.294117647	0.422955
DCM	TTN	c.24160A>T	p.Ile8054Leu	missense_variant	2	0.00511509	0.021406
DCM	TTN	c.23538C>G	p.Phe7846Leu	missense_variant	1	0.002557545	0.020492
DCM	TTN	c.23455G>C	p.Glu7819Gln	missense_variant	1	0.002557545	0.000253
DCM	TTN	c.23232C>G	p.Asn7744Lys	missense_variant	8	0.020460358	0.017655
DCM	TTN	c.22978A>G	p.Ile7660Val	missense_variant	1	0.002557545	0
DCM	TTN	c.22978A>C	p.Ile7660Leu	missense_variant	1	0.002557545	0

DCM	TTN	c.22786G>C	p.Asp7596His	missense_variant	19	0.04859335	0.044353
DCM	TTN	c.22634G>A	p.Arg7545Gln	missense_variant	1	0.002557545	0.002407
DCM	TTN	c.22384G>C	p.Asp7462His	missense_variant	117	0.299232737	0.423007
DCM	TTN	c.21779C>A	p.Ser7260Tyr	missense_variant	6	0.015345269	0.025641
DCM	TTN	c.21521C>T	p.Ala7174Val	missense_variant	1	0.002557545	3.00E-06
DCM	TTN	c.21288T>A	p.Asp7096Glu	missense_variant	2	0.00511509	0
DCM	TTN	c.21173G>A	p.Gly7058Asp	missense_variant	16	0.040920716	0.008146
DCM	TTN	c.21106G>A	p.Asp7036Asn	missense_variant	10	0.025575448	0.050807
DCM	TTN	c.21044C>T	p.Ala7015Val	missense_variant	7	0.017902813	0.118524
DCM	TTN	c.20896A>G	p.Thr6966Ala	missense_variant	1	0.002557545	0
DCM	TTN	c.20861C>T	p.Ala6954Val	missense_variant	18	0.046035806	0.179989
DCM	TTN	c.20630T>C	p.Ile6877Thr	missense_variant	1	0.002557545	0.002985
DCM	TTN	c.20602G>A	p.Gly6868Arg	missense_variant	8	0.020460358	0.017285
DCM	TTN	c.20175A>G	p.Ile6725Met	missense_variant	1	0.002557545	0.020346
DCM	TTN	c.20147T>A	p.Met6716Lys	missense_variant	1	0.002557545	0.017834
DCM	TTN	c.20108G>A	p.Arg6703Gln	missense_variant	2	0.00511509	0.000127
DCM	TTN	c.20041G>A	p.Ala6681Thr	missense_variant	1	0.002557545	3.00E-06
DCM	TTN	c.19976C>T	p.Thr6659Met	missense_variant	12	0.030690537	0.040355
DCM	TTN	c.19301G>A	p.Ser6434Asn	missense_variant	30	0.076726343	0.182113
DCM	TTN	c.19276G>A	p.Val6426Ile	missense_variant	1	0.002557545	0
DCM	TTN	c.19204A>G	p.Met6402Val	missense_variant	8	0.020460358	0.048498
DCM	TTN	c.19016A>G	p.Tyr6339Cys	missense_variant	1	0.002557545	0.000155
DCM	TTN	c.19004A>G	p.Asp6335Gly	missense_variant	2	0.00511509	0.030254
DCM	TTN	c.18856G>A	p.Val6286Ile	missense_variant	1	0.002557545	0.005269
DCM	TTN	c.18832G>A	p.Gly6278Ser	missense_variant	1	0.002557545	4.50E-05
DCM	TTN	c.18824A>G	p.Asn6275Ser	missense_variant	1	0.002557545	0.020939
DCM	TTN	c.18776C>G	p.Thr6259Ser	missense_variant	14	0.035805627	0.002908

DCM	TTN	c.18550G>A	p.Ala6184Thr	missense_variant	7	0.017902813	0.000482
DCM	TTN	c.18427G>A	p.Gly6143Arg	missense_variant	2	0.00511509	1.10E-05
DCM	TTN	c.18325A>G	p.Lys6109Glu	missense_variant	2	0.00511509	0.003066
DCM	TTN	c.18007A>C	p.Ser6003Arg	missense_variant	1	0.002557545	0
DCM	TTN	c.17686G>A	p.Glu5896Lys	missense_variant	1	0.002557545	0.001364
DCM	TTN	c.17312C>G	p.Thr5771Ser	missense_variant	3	0.007672634	0.018623
DCM	TTN	c.17048A>G	p.Tyr5683Cys	missense_variant	1	0.002557545	0.007722
DCM	TTN	c.16303G>A	p.Val5435Met	missense_variant	2	0.00511509	0.008117
DCM	TTN	c.16091G>A	p.Arg5364His	missense_variant	1	0.002557545	0.000305
DCM	TTN	c.16040C>T	p.Thr5347Ile	missense_variant	2	0.00511509	0
DCM	TTN	c.15986G>A	p.Gly5329Asp	missense_variant	2	0.00511509	0.000604
DCM	TTN	c.15584A>G	p.Glu5195Gly	missense_variant	1	0.002557545	0.009511
DCM	TTN	c.15211A>G	p.Ile5071Val	missense_variant	1	0.002557545	7.00E-06
DCM	TTN	c.15196A>G	p.Ser5066Gly	missense_variant	1	0.002557545	7.00E-06
DCM	TTN	c.15178G>C	p.Val5060Leu	missense_variant	1	0.002557545	0.00701
DCM	TTN	c.14870C>G	p.Thr4957Ser	missense_variant	15	0.038363171	0.004538
DCM	TTN	c.14813T>C	p.Phe4938Ser	missense_variant	1	0.002557545	0.000245
DCM	TTN	c.14726G>A	p.Cys4909Tyr	missense_variant	1	0.002557545	0
DCM	TTN	c.14525G>A	p.Arg4842Lys	missense_variant	51	0.130434783	0.247743
DCM	TTN	c.14307G>C	p.Glu4769Asp	missense_variant	1	0.002557545	0
DCM	TTN	c.14050G>A	p.Gly4684Arg	missense_variant	1	0.002557545	9.30E-05
DCM	TTN	c.14002A>G	p.Thr4668Ala	missense_variant	1	0.002557545	4.40E-05
DCM	TTN	c.13969A>C	p.Asn4657His	missense_variant	1	0.002557545	0.01656
DCM	TTN	c.13940A>G	p.Asp4647Gly	missense_variant	1	0.002557545	1.20E-05
DCM	TTN	c.13859G>A	p.Gly4620Asp	missense_variant	1	0.002557545	0.020996
DCM	TTN	c.13800A>C	p.Leu4600Phe	missense_variant	4	0.010230179	0.23682
DCM	TTN	c.13724A>G	p.Glu4575Gly	missense_variant	1	0.002557545	5.20E-05

DCM	TTN	c.13706G>A	p.Ser4569Asn	missense_variant	1	0.002557545	0.01133
DCM	TTN	c.13594A>C	p.Thr4532Pro	missense_variant	4	0.010230179	0.236849
DCM	TTN	c.13520T>C	p.Met4507Thr	missense_variant	1	0.002557545	0.001129
DCM	TTN	c.13228G>A	p.Glu4410Lys	missense_variant	2	0.00511509	2.30E-05
DCM	TTN	c.13124G>C	p.Arg4375Thr	missense_variant	1	0.002557545	0
DCM	TTN	c.12580A>T	p.Ile4194Phe	missense_variant	1	0.002557545	0.005533
DCM	TTN	c.12235A>G	p.Ile4079Val	missense_variant	24	0.061381074	0.039188
DCM	TTN	c.12233C>T	p.Thr4078Ile	missense_variant	2	0.00511509	0.012187
DCM	TTN	c.12066T>G	p.Cys4022Trp	missense_variant	1	0.002557545	0
DCM	TTN	c.11996A>G	p.Asn3999Ser	missense_variant	1	0.002557545	0.000177
DCM	TTN	c.11974G>A	p.Gly3992Ser	missense_variant	2	0.00511509	1.20E-05
DCM	TTN	c.11788G>A	p.Glu3930Lys	missense_variant	3	0.007672634	0.000433
DCM	TTN	c.11719C>G	p.Leu3907Val	missense_variant	4	0.010230179	0.035877
DCM	TTN	c.11422C>T	p.Pro3808Ser	missense_variant	82	0.20971867	0.440587
DCM	TTN	c.11399T>A	p.Leu3800His	missense_variant	1	0.002557545	0
DCM	TTN	c.11252G>A	p.Gly3751Asp	missense_variant ,splice_region_variant	389	0.99488491	0.925371
DCM	TTN	c.10850C>T	p.Ser3617Phe	missense_variant	8	0.020460358	0.05316
DCM	TTN	c.10770G>C	p.Glu3590Asp	missense_variant	4	0.010230179	0.000201
DCM	TTN	c.10726G>A	p.Ala3576Thr	missense_variant	391	1	0.99508
DCM	TTN	c.10700G>A	p.Ser3567Asn	missense_variant	11	0.028132992	0.019637
DCM	TTN	c.10430A>G	p.His3477Arg	missense_variant	1	0.002557545	0
DCM	TTN	c.10378C>G	p.Pro3460Ala	missense_variant	1	0.002557545	0.001257
DCM	TTN	c.10256G>A	p.Ser3419Asn	missense_variant	389	0.99488491	0.92547
DCM	TTN	c.10100G>A	p.Arg3367Gln	missense_variant	1	0.002557545	0.015041
DCM	TTN	c.9826G>A	p.Glu3276Lys	missense_variant	1	0.002557545	0
DCM	TTN	c.9781G>A	p.Val3261Met	missense_variant	389	0.99488491	0.925392

DCM	TTN	c.9461A>G	p.Lys3154Arg	missense_variant	24	0.061381074	0.181724
DCM	TTN	c.9359G>A	p.Arg3120Gln	missense_variant	1	0.002557545	0.005126
DCM	TTN	c.9167G>A	p.Arg3056His	missense_variant	1	0.002557545	0.00013
DCM	TTN	c.9077A>T	p.Asn3026Ile	missense_variant	2	0.00511509	0.005571
DCM	TTN	c.8492G>A	p.Ser2831Asn	missense_variant	5	0.012787724	0.235921
DCM	TTN	c.8467G>T	p.Val2823Phe	missense_variant	12	0.030690537	0.021489
DCM	TTN	c.8165A>G	p.Gln2722Arg	missense_variant	1	0.002557545	2.60E-05
DCM	TTN	c.7979C>T	p.Pro2660Leu	missense_variant	1	0.002557545	0
DCM	TTN	c.7830G>C	p.Met2610Ile	missense_variant	4	0.010230179	0.234515
DCM	TTN	c.7174G>A	p.Gly2392Ser	missense_variant	32	0.081841432	0.183635
DCM	TTN	c.7061G>A	p.Arg2354His	missense_variant	1	0.002557545	0.00853
DCM	TTN	c.6950G>A	p.Arg2317His	missense_variant	1	0.002557545	0.000115
DCM	TTN	c.6877G>C	p.Glu2293Gln	missense_variant	1	0.002557545	0
DCM	TTN	c.6353T>C	p.Ile2118Thr	missense_variant	2	0.00511509	0.014518
DCM	TTN	c.6292C>T	p.Arg2098Trp	missense_variant	1	0.002557545	2.30E-05
DCM	TTN	c.5993G>A	p.Arg1998His	missense_variant	1	0.002557545	0.006748
DCM	TTN	c.5668C>T	p.Arg1890Cys	missense_variant	1	0.002557545	0.001381
DCM	TTN	c.5644C>T	p.Arg1882Cys	missense_variant	1	0.002557545	1.20E-05
DCM	TTN	c.5641A>G	p.Ile1881Val	missense_variant	1	0.002557545	0
DCM	TTN	c.5582G>A	p.Arg1861His	missense_variant	3	0.007672634	9.50E-05
DCM	TTN	c.5231C>T	p.Pro1744Leu	missense_variant	1	0.002557545	0.034504
DCM	TTN	c.4961C>T	p.Pro1654Leu	missense_variant	1	0.002557545	0
DCM	TTN	c.4739C>T	p.Thr1580Met	missense_variant	19	0.04859335	0.016448
DCM	TTN	c.4715G>A	p.Arg1572Gln	missense_variant	4	0.010230179	0.234926
DCM	TTN	c.4671G>A	p.Met1557Ile	missense_variant	2	0.00511509	0.00278
DCM	TTN	c.4630A>G	p.Ile1544Val	missense_variant	32	0.081841432	0.032493
DCM	TTN	c.4322G>C	p.Arg1441Pro	missense_variant	5	0.012787724	0.015652

DCM	TTN	c.4291C>T	p.Arg1431Trp	missense_variant	2	0.00511509	0.000406
DCM	TTN	c.4246C>T	p.Arg1416Cys	missense_variant	3	0.007672634	0.050161
DCM	TTN	c.4177A>G	p.Ile1393Val	missense_variant	8	0.020460358	0.127589
DCM	TTN	c.4063C>A	p.Leu1355Ile	missense_variant	2	0.00511509	0
DCM	TTN	c.4034G>A	p.Gly1345Asp	missense_variant	1	0.002557545	0.008044
DCM	TTN	c.3884C>T	p.Ser1295Leu	missense_variant	391	1	0.98516
DCM	TTN	c.3668C>T	p.Ala1223Val	missense_variant	2	0.00511509	0.011496
DCM	TTN	c.3601A>G	p.Lys1201Glu	missense_variant	382	0.976982097	0.864022
DCM	TTN	c.3469G>A	p.Val1157Ile	missense_variant	2	0.00511509	5.40E-05
DCM	TTN	c.3319G>A	p.Gly1107Ser	missense_variant	1	0.002557545	1.20E-05
DCM	TTN	c.3295G>A	p.Val1099Met	missense_variant	1	0.002557545	2.30E-05
DCM	TTN	c.3100G>A	p.Val1034Met	missense_variant,splice_region_variant	2	0.00511509	0.000925
DCM	TTN	c.3031G>A	p.Gly1011Arg	missense_variant	1	0.002557545	2.30E-05
DCM	TTN	c.2996G>A	p.Arg999His	missense_variant	1	0.002557545	0.000107
DCM	TTN	c.2765G>A	p.Arg922His	missense_variant	9	0.023017903	0.008196
DCM	TTN	c.2744G>C	p.Arg915Pro	missense_variant	1	0.002557545	7.00E-06
DCM	TTN	c.2432C>T	p.Thr811Ile	missense_variant	173	0.442455243	0.23494
DCM	TTN	c.2386G>A	p.Asp796Asn	missense_variant	1	0.002557545	7.50E-05
DCM	TTN	c.1492G>A	p.Val498Ile	missense_variant	9	0.023017903	0.021567
DCM	TTN	c.1133G>A	p.Gly378Glu	missense_variant	1	0.002557545	0
DCM	TTN	c.1079G>C	p.Arg360Thr	missense_variant	2	0.00511509	0.021492
DCM	TTN	c.1003G>A	p.Val335Met	missense_variant	3	0.007672634	0.041025
DCM	TTN	c.982C>T	p.Arg328Cys	missense_variant	42	0.10741688	0.588116
DCM	TTN	c.970C>T	p.Pro324Ser	missense_variant	1	0.002557545	0.007394
DCM	TTN	c.908C>T	p.Pro303Leu	missense_variant	1	0.002557545	1.00E-05
DCM	TTN	c.542G>A	p.Ser181Asn	missense_variant	2	0.00511509	0.056194

DCM	TTN	c.427G>A	p.Glu143Lys	missense_variant	1	0.002557545	2.30E-05
DCM	TTN	c.266C>G	p.Ala89Gly	missense_variant	1	0.002557545	0.000212
DCM	TTN	c.227G>C	p.Gly76Ala	missense_variant	1	0.002557545	0.000102
DCM	TTN	c.178G>T	p.Asp60Tyr	missense_variant	16	0.040920716	0.067107
DCM	TTN	c.67G>A	p.Ala23Thr	missense_variant	1	0.002557545	0
HCM	MYL3	c.532G>A	p.Asp178Asn	missense_variant	3	0.007672634	0.001157
HCM	MYL3	c.530A>G	p.Glu177Gly	missense_variant	2	0.00511509	4.40E-05
syndromic gene (associated with isolated LVH)	RAF1	c.1913C>T	p.Thr638Met	missense_variant	1	0.002557545	2.60E-05
syndromic gene (associated with isolated LVH)	RAF1	c.1516A>G	p.Thr506Ala	missense_variant	1	0.002557545	0
syndromic gene (associated with isolated LVH)	RAF1	c.770C>T	p.Ser257Leu	missense_variant	1	0.002557545	0
syndromic gene (associated with isolated LVH)	RAF1	c.723C>G	p.Asn241Lys	missense_variant	1	0.002557545	0
syndromic gene (associated with isolated LVH)	RAF1	c.601A>G	p.Ile201Val	missense_variant	1	0.002557545	1.20E-05
syndromic gene (associated with isolated LVH)	RAF1	c.125C>T	p.Ala42Val	missense_variant	2	0.00511509	0.00028
syndromic gene (associated with isolated LVH)	RAF1	c.124G>A	p.Ala42Thr	missense_variant	2	0.00511509	0.00028
syndromic gene (associated with isolated LVH)	RAF1	c.122G>A	p.Arg41Gln	missense_variant	2	0.00511509	0.00029

DCM	SCN5A	c.5972G>A	p.Arg1991Gln	missense_variant	2	0.00511509	0
DCM	SCN5A	c.5494C>G	p.Gln1832Glu	missense_variant	1	0.002557545	0.000397
DCM	SCN5A	c.3883G>A	p.Glu1295Lys	missense_variant	1	0.002557545	0
DCM	SCN5A	c.3878T>C	p.Phe1293Ser	missense_variant	1	0.002557545	8.00E-04
DCM	SCN5A	c.3835G>A	p.Val1279Ile	missense_variant	1	0.002557545	9.50E-05
DCM	SCN5A	c.3662C>T	p.Ala1221Val	missense_variant	1	0.002557545	2.70E-05
DCM	SCN5A	c.2944T>C	p.Cys982Arg	missense_variant	1	0.002557545	0.000321
DCM	SCN5A	c.1901C>T	p.Ser634Leu	missense_variant	1	0.002557545	8.50E-05
DCM	SCN5A	c.1715C>T	p.Ala572Val	missense_variant	1	0.002557545	0.00145
DCM	SCN5A	c.1714G>T	p.Ala572Ser	missense_variant	1	0.002557545	0.001449
DCM	SCN5A	c.1700T>A	p.Leu567Gln	missense_variant	1	0.002557545	0
DCM	SCN5A	c.1673A>G	p.His558Arg	missense_variant	182	0.465473146	0.28512
DCM	SCN5A	c.1604G>A	p.Arg535Gln	missense_variant	1	0.002557545	7.00E-06
DCM	SCN5A	c.1571C>A	p.Ser524Tyr	missense_variant	4	0.010230179	0.03464
DCM	SCN5A	c.1558A>G	p.Met520Val	missense_variant	1	0.002557545	8.90E-05
DCM	SCN5A	c.1550G>A	p.Arg517Lys	missense_variant	2	0.00511509	0
DCM	SCN5A	c.1441C>T	p.Arg481Trp	missense_variant	1	0.002557545	0.009996
DCM	SCN5A	c.1153G>A	p.Ala385Thr	missense_variant	2	0.00511509	3.00E-06
DCM	SCN5A	c.182C>T	p.Ser61Phe	missense_variant	1	0.002557545	0
DCM	SCN5A	c.100C>T	p.Arg34Cys	missense_variant	26	0.066496164	0.096916
DCM	SCN5A	c.52C>T	p.Arg18Trp	missense_variant	1	0.002557545	0.00136
DCM	SCN5A	c.44G>C	p.Arg15Thr	missense_variant	1	0.002557545	5.70E-05
DCM	DSP	c.88G>A	p.Val30Met	missense_variant	2	0.00511509	0.003687
DCM	DSP	c.485G>A	p.Arg162His	missense_variant	1	0.002557545	6.40E-05
DCM	DSP	c.913A>T	p.Ile305Phe	missense_variant	14	0.035805627	0.038608
DCM	DSP	c.1373A>C	p.Asn458Thr	missense_variant	1	0.002557545	0
DCM	DSP	c.1778A>G	p.Asn593Ser	missense_variant	1	0.002557545	0.000943

DCM	DSP	c.2330T>A	p.Ile777Asn	missense_variant	1	0.002557545	0
DCM	DSP	c.2468C>T	p.Ser823Leu	missense_variant	1	0.002557545	0.000244
DCM	DSP	c.2569G>A	p.Gly857Ser	missense_variant	1	0.002557545	3.40E-05
DCM	DSP	c.2606G>A	p.Arg869Lys	missense_variant	2	0.00511509	0
DCM	DSP	c.2684A>G	p.Tyr895Cys	missense_variant	7	0.017902813	0.00038
DCM	DSP	c.2723G>A	p.Arg908His	missense_variant	15	0.038363171	0.001332
DCM	DSP	c.2774G>A	p.Arg925Gln	missense_variant	3	0.007672634	0.000459
DCM	DSP	c.2815G>A	p.Gly939Ser	missense_variant	10	0.025575448	0.037308
DCM	DSP	c.3071T>G	p.Leu1024Trp	missense_variant	1	0.002557545	0
DCM	DSP	c.3465G>C	p.Trp1155Cys	missense_variant	1	0.002557545	0
DCM	DSP	c.3760G>A	p.Val1254Ile	missense_variant	1	0.002557545	3.50E-05
DCM	DSP	c.3802_3807dupAGGCGA	p.Arg1268_Arg1269dup	inframe_insertion	1	0.002557545	0
DCM	DSP	c.3815A>G	p.Glu1272Gly	missense_variant	1	0.002557545	0
DCM	DSP	c.3862A>C	p.Lys1288Gln	missense_variant	2	0.00511509	0.000295
DCM	DSP	c.4141A>T	p.Thr1381Ser	missense_variant	2	0.00511509	0.008152
DCM	DSP	c.4387G>A	p.Val1463Ile	missense_variant	1	0.002557545	1.70E-05
DCM	DSP	c.4455G>T	p.Arg1485Ser	missense_variant	1	0.002557545	0.018243
DCM	DSP	c.4535A>G	p.Tyr1512Cys	missense_variant	83	0.212276215	0.287763
DCM	DSP	c.4578C>A	p.Asn1526Lys	missense_variant	17	0.043478261	0.147262
DCM	DSP	c.5155A>G	p.Met1719Val	missense_variant	1	0.002557545	0
DCM	DSP	c.5196_5198delTGA	p.Asp1733del	inframe_deletion	1	0.002557545	0
DCM	DSP	c.5213G>A	p.Arg1738Gln	missense_variant	121	0.309462916	0.322134
DCM	DSP	c.5218G>A	p.Glu1740Lys	missense_variant	12	0.030690537	0.001727
DCM	DSP	c.5324G>T	p.Arg1775Ile	missense_variant	1	0.002557545	0.000177
DCM	DSP	c.5647T>C	p.Ser1883Pro	missense_variant	1	0.002557545	0
DCM	DSP	c.5711C>T	p.Ala1904Val	missense_variant	1	0.002557545	0

DCM	DSP	c.5726T>C	p.Ile1909Thr	missense_variant	1	0.002557545	0
DCM	DSP	c.6208G>A	p.Asp2070Asn	missense_variant	6	0.015345269	0.005444
DCM	DSP	c.6550G>A	p.Val2184Met	missense_variant	1	0.002557545	2.80E-05
DCM	DSP	c.7175A>G	p.Tyr2392Cys	missense_variant	1	0.002557545	0
DCM	DSP	c.7552G>A	p.Val2518Ile	missense_variant	1	0.002557545	2.30E-05
DCM	DSP	c.7745T>G	p.Phe2582Cys	missense_variant	2	0.00511509	0
DCM	DSP	c.8206C>T	p.Leu2736Phe	missense_variant	1	0.002557545	0
DCM	DSP	c.8237G>C	p.Ser2746Thr	missense_variant	1	0.002557545	0
DCM	DSP	c.8343A>T	p.Leu2781Phe	missense_variant	1	0.002557545	0
DCM	DSP	c.8590T>C	p.Phe2864Leu	missense_variant	1	0.002557545	2.20E-05
syndromic gene (associated with isolated LVH)	PRKAG2	c.1475T>A	p.Ile492Asn	missense_variant	5	0.012787724	2.20E-05
syndromic gene (associated with isolated LVH)	PRKAG2	c.745G>A	p.Glu249Lys	missense_variant	1	0.002557545	0
syndromic gene (associated with isolated LVH)	PRKAG2	c.359G>A	p.Arg120His	missense_variant	1	0.002557545	0.000575
syndromic gene (associated with isolated LVH)	PRKAG2	c.298G>A	p.Gly100Ser	missense_variant	10	0.025575448	0.033119
syndromic gene (associated with isolated LVH)	PRKAG2	c.59G>T	p.Ser20Ile	missense_variant	1	0.002557545	0.063676
syndromic gene (associated with isolated LVH)	FHL1	c.823G>A	p.Asp275Asn	missense_variant	1	0.002557545	0.017913
syndromic gene (associated with isolated LVH)	GLA	c.937G>T	p.Asp313Tyr	missense_variant	1	0.002557545	0.004054

syndromic gene (associated with isolated LVH)	GLA	c.525C>A	p.Asp175Glu	missense_variant	1	0.002557545	0
syndromic gene (associated with isolated LVH)	LAMP2	c.755T>G	p.Ile252Ser	missense_variant	2	0.00511509	0.001639
DCM & HCM	MYH7	c.2337_2341dupGAGGC	p.Leu781ArgfsTer3	frameshift_variant	1	0.002557545	0
DCM	TTN	c.104524delT	p.Ser34842ProfsTer9	frameshift_variant	1	0.002557545	0
DCM	TTN	c.37362dupC	p.Thr12455HisfsTer18	frameshift_variant	1	0.002557545	0
DCM	TTN	c.32471-1G>A	-	splice_acceptor_variant	2	0.00511509	2.70E-05
DCM	TTN	c.25639+1G>T	-	splice_donor_variant	2	0.00511509	0
DCM	TTN	c.19417A>T	p.Arg6473Ter	stop_gained	1	0.002557545	0
DCM	LMNA	c.51C>T	p.Ser17%3D	synonymous_variant	11	0.028132992	0.013387
DCM	LMNA	c.612G>A	p.Leu204%3D	synonymous_variant	15	0.038363171	0.0252
DCM	LMNA	c.861T>C	p.Ala287%3D	synonymous_variant	143	0.3657289	0.449458
DCM	LMNA	c.1011G>C	p.Leu337%3D	synonymous_variant	1	0.002557545	0
DCM	LMNA	c.1338T>C	p.Asp446%3D	synonymous_variant	152	0.388746803	0.629349
DCM & HCM	TNNT2	c.444G>C	p.Arg148%3D	synonymous_variant	21	0.05370844	0.024166
DCM & HCM	TNNT2	c.318C>T	p.Ile106%3D	synonymous_variant	343	0.877237852	0.808382

DCM & HCM	TNNT2	c.207G>A	p.Ser69%3D	synonymous_variant	85	0.217391304	0.149585
DCM	BAG3	c.231G>A	p.Pro77%3D	synonymous_variant	6	0.015345269	0.014281
DCM	BAG3	c.573C>T	p.Ser191%3D	synonymous_variant	1	0.002557545	2.20E-05
DCM	BAG3	c.693G>A	p.Thr231%3D	synonymous_variant	1	0.002557545	0.000244
DCM	BAG3	c.795G>A	p.Pro265%3D	synonymous_variant	1	0.002557545	4.40E-05
DCM	BAG3	c.1002T>G	p.Pro334%3D	synonymous_variant	57	0.145780051	0.259735
DCM	BAG3	c.1296A>G	p.Val432%3D	synonymous_variant	376	0.961636829	0.78015
DCM	RBM20	c.90G>A	p.Arg30%3D	synonymous_variant	142	0.363171355	0.394893
DCM	RBM20	c.327T>C	p.Ala109%3D	synonymous_variant	1	0.002557545	0
DCM	RBM20	c.447T>C	p.His149%3D	synonymous_variant	1	0.002557545	0
DCM	RBM20	c.1002A>T	p.Thr334%3D	synonymous_variant	1	0.002557545	0
DCM	RBM20	c.1378T>C	p.Leu460%3D	synonymous_variant	2	0.00511509	0.000172
DCM	RBM20	c.1659G>A	p.Ser553%3D	synonymous_variant	1	0.002557545	0.000404
DCM	RBM20	c.1914G>A	p.Pro638%3D	synonymous_variant	2	0.00511509	0.058767
DCM	RBM20	c.3144C>T	p.Ser1048%3D	synonymous_variant	2	0.00511509	0.049807
DCM	VCL	c.339G>A	p.Arg113%3D	synonymous_variant	21	0.05370844	0.213748

DCM	VCL	c.768T>C	p.Asp256%3D	synonymous_variant	23	0.058823529	0.214835
DCM	VCL	c.945C>A	p.Gly315%3D	synonymous_variant	24	0.061381074	0.089594
DCM	VCL	c.1317T>C	p.Ser439%3D	synonymous_variant	9	0.023017903	0.006056
DCM	VCL	c.1407C>T	p.Ala469%3D	synonymous_variant	48	0.122762148	0.207953
DCM	VCL	c.1506G>A	p.Arg502%3D	synonymous_variant	48	0.122762148	0.207566
DCM	VCL	c.1671C>T	p.Asp557%3D	synonymous_variant	9	0.023017903	0.016409
DCM	VCL	c.2368T>C	p.Leu790%3D	synonymous_variant	1	0.002557545	3.00E-06
DCM	VCL	c.2388G>A	p.Pro796%3D	synonymous_variant	308	0.787723785	0.576651
DCM	VCL	c.2814C>G	p.Gly938%3D	synonymous_variant	389	0.99488491	0.768883
DCM	VCL	c.3102G>A	p.Lys1034%3D	synonymous_variant	1	0.002557545	5.00E-05
DCM	VCL	c.3189C>T	p.Leu1063%3D	synonymous_variant	1	0.002557545	1.20E-05
HCM	MYBPC3	c.3780C>T	p.Gly1260%3D	synonymous_variant	1	0.002557545	3.00E-06
HCM	MYBPC3	c.3741C>T	p.Asp1247%3D	synonymous_variant	1	0.002557545	0
HCM	MYBPC3	c.3657G>T	p.Leu1219%3D	synonymous_variant	5	0.012787724	1.20E-05
HCM	MYBPC3	c.3288G>A	p.Glu1096%3D	synonymous_variant	196	0.501278772	0.688534
HCM	MYBPC3	c.3270G>A	p.Gln1090%3D	synonymous_variant	1	0.002557545	0

HCM	MYBPC3	c.2961C>T	p.Val987%3D	synonymous_variant	1	0.002557545	7.90E-05
HCM	MYBPC3	c.2547C>T	p.Val849%3D	synonymous_variant	25	0.063938619	0.115371
HCM	MYBPC3	c.2535C>G	p.Arg845%3D	synonymous_variant	2	0.00511509	0
HCM	MYBPC3	c.2511C>T	p.Ile837%3D	synonymous_variant	1	0.002557545	2.20E-05
HCM	MYBPC3	c.2064G>A	p.Thr688%3D	synonymous_variant	1	0.002557545	3.90E-05
HCM	MYBPC3	c.1566G>A	p.Ala522%3D	synonymous_variant	3	0.007672634	0.000259
HCM	MYBPC3	c.1446G>A	p.Ala482%3D	synonymous_variant	1	0.002557545	0
HCM	MYBPC3	c.1282T>C	p.Leu428%3D	synonymous_variant	2	0.00511509	0.000184
HCM	MYBPC3	c.786C>T	p.Thr262%3D	synonymous_variant	62	0.158567775	0.123443
HCM	MYBPC3	c.537C>T	p.Ala179%3D	synonymous_variant	1	0.002557545	0.079895
HCM	MYBPC3	c.492C>T	p.Gly164%3D	synonymous_variant	13	0.033248082	0.030587
HCM	MYBPC3	c.471C>T	p.Phe157%3D	synonymous_variant	1	0.002557545	0.000627
syndromic gene (associated with isolated LVH)	CACNA1C	c.171C>T	p.Asp57%3D	synonymous_variant	20	0.051150895	0.035216
syndromic gene (associated with isolated LVH)	CACNA1C	c.213G>A	p.Ala71%3D	synonymous_variant	14	0.035805627	0.039398
syndromic gene (associated with isolated LVH)	CACNA1C	c.237G>A	p.Thr79%3D	synonymous_variant	1	0.002557545	1.70E-05

syndromic gene (associated with isolated LVH)	CACNA1C	c.363C>T	p.Val121%3D	synonymous_variant	1	0.002557545	3.00E-06
syndromic gene (associated with isolated LVH)	CACNA1C	c.459C>T	p.Asn153%3D	synonymous_variant	2	0.00511509	4.70E-05
syndromic gene (associated with isolated LVH)	CACNA1C	c.522G>A	p.Ala174%3D	synonymous_variant	124	0.31713555	0.206989
syndromic gene (associated with isolated LVH)	CACNA1C	c.771C>T	p.Val257%3D	synonymous_variant	1	0.002557545	0.030004
syndromic gene (associated with isolated LVH)	CACNA1C	c.846C>T	p.Tyr282%3D	synonymous_variant	3	0.007672634	0.000267
syndromic gene (associated with isolated LVH)	CACNA1C	c.906G>A	p.Glu302%3D	synonymous_variant	2	0.00511509	4.40E-05
syndromic gene (associated with isolated LVH)	CACNA1C	c.1053C>T	p.Phe351%3D	synonymous_variant	1	0.002557545	3.90E-05
syndromic gene (associated with isolated LVH)	CACNA1C	c.1557C>T	p.Ala519%3D	synonymous_variant	1	0.002557545	6.30E-05
syndromic gene (associated with isolated LVH)	CACNA1C	c.1707A>G	p.Ala569%3D	synonymous_variant	1	0.002557545	0.001644
syndromic gene (associated with isolated LVH)	CACNA1C	c.2067C>T	p.Phe689%3D	synonymous_variant	1	0.002557545	0.000239
syndromic gene (associated with isolated LVH)	CACNA1C	c.2391G>A	p.Gly797%3D	synonymous_variant	1	0.002557545	0.000289

syndromic gene (associated with isolated LVH)	CACNA1C	c.2436C>T	p.Asp812%3D	synonymous_variant	121	0.309462916	0.27936
syndromic gene (associated with isolated LVH)	CACNA1C	c.2766G>A	p.Pro922%3D	synonymous_variant	1	0.002557545	0.001178
syndromic gene (associated with isolated LVH)	CACNA1C	c.3114G>C	p.Leu1038%3D	synonymous_variant	3	0.007672634	0.017386
syndromic gene (associated with isolated LVH)	CACNA1C	c.3387G>A	p.Thr1129%3D	synonymous_variant	4	0.010230179	0.014603
syndromic gene (associated with isolated LVH)	CACNA1C	c.3459C>T	p.Ile1153%3D	synonymous_variant	1	0.002557545	1.90E-05
syndromic gene (associated with isolated LVH)	CACNA1C	c.3576C>T	p.Tyr1192%3D	synonymous_variant	1	0.002557545	3.90E-05
syndromic gene (associated with isolated LVH)	CACNA1C	c.3780C>A	p.Gly1260%3D	synonymous_variant	4	0.010230179	0.003927
syndromic gene (associated with isolated LVH)	CACNA1C	c.3786C>T	p.Phe1262%3 D	synonymous_variant	172	0.439897698	0.343489
syndromic gene (associated with isolated LVH)	CACNA1C	c.3789C>T	p.Thr1263%3D	synonymous_variant	1	0.002557545	0
syndromic gene (associated with isolated LVH)	CACNA1C	c.4038C>T	p.Ile1346%3D	synonymous_variant	23	0.058823529	0.065977
syndromic gene (associated with isolated LVH)	CACNA1C	c.4122C>T	p.Tyr1374%3D	synonymous_variant	3	0.007672634	0.000547

syndromic gene (associated with isolated LVH)	CACNA1C	c.4485T>C	p.Asp1495%3D	synonymous_variant	23	0.058823529	0.130514
syndromic gene (associated with isolated LVH)	CACNA1C	c.5064C>T	p.Ser1688%3D	synonymous_variant	1	0.002557545	0.015539
syndromic gene (associated with isolated LVH)	CACNA1C	c.5097C>T	p.Ala1699%3D	synonymous_variant	19	0.04859335	0.00892
syndromic gene (associated with isolated LVH)	CACNA1C	c.5118C>T	p.His1706%3D	synonymous_variant	1	0.002557545	0
syndromic gene (associated with isolated LVH)	CACNA1C	c.5139C>T	p.Asp1713%3D	synonymous_variant	5	0.012787724	0.014545
syndromic gene (associated with isolated LVH)	CACNA1C	c.5241G>A	p.Lys1747%3D	synonymous_variant	1	0.002557545	0
syndromic gene (associated with isolated LVH)	CACNA1C	c.5277G>A	p.Ser1759%3D	synonymous_variant	1	0.002557545	3.90E-05
syndromic gene (associated with isolated LVH)	CACNA1C	c.5292C>T	p.Asn1764%3D	synonymous_variant	31	0.079283887	0.063526
syndromic gene (associated with isolated LVH)	CACNA1C	c.5361G>A	p.Thr1787%3D	synonymous_variant	271	0.693094629	0.718256
syndromic gene (associated with isolated LVH)	CACNA1C	c.5451C>T	p.His1817%3D	synonymous_variant	3	0.007672634	0.000759
syndromic gene (associated with isolated LVH)	CACNA1C	c.5568A>G	p.Thr1856%3D	synonymous_variant	4	0.010230179	0

syndromic gene (associated with isolated LVH)	CACNA1C	c.5604A>G	p.Gln1868%3D	synonymous_variant	17	0.043478261	0.02697
syndromic gene (associated with isolated LVH)	CACNA1C	c.5649G>A	p.Pro1883%3D	synonymous_variant	22	0.056265985	0.038472
HCM	MYL2	c.132T>C	p.Ile44%3D	synonymous_variant	42	0.10741688	0.231321
syndromic gene (associated with isolated LVH)	PTPN11	c.255C>T	p.His85%3D	synonymous_variant	12	0.030690537	0.073292
syndromic gene (associated with isolated LVH)	PTPN11	c.558G>T	p.Arg186%3D	synonymous_variant	1	0.002557545	0.002389
syndromic gene (associated with isolated LVH)	PTPN11	c.1242G>A	p.Thr414%3D	synonymous_variant	1	0.002557545	0
DCM & HCM	MYH7	c.5727G>A	p.Arg1909%3D	synonymous_variant	1	0.002557545	1.70E-05
DCM & HCM	MYH7	c.5106G>A	p.Ala1702%3D	synonymous_variant	104	0.265984655	0.220639
DCM & HCM	MYH7	c.4716C>T	p.Ile1572%3D	synonymous_variant	8	0.020460358	0.045495
DCM & HCM	MYH7	c.4566T>C	p.Thr1522%3D	synonymous_variant	9	0.023017903	0.030323
DCM & HCM	MYH7	c.4494C>T	p.Phe1498%3 D	synonymous_variant	3	0.007672634	0
DCM & HCM	MYH7	c.4410G>A	p.Ser1470%3D	synonymous_variant	1	0.002557545	0.000107
DCM & HCM	MYH7	c.4290G>T	p.Val1430%3D	synonymous_variant	1	0.002557545	4.10E-05
DCM & HCM	MYH7	c.4239G>A	p.Ser1413%3D	synonymous_variant	1	0.002557545	0.005727

DCM & HCM	MYH7	c.3612C>T	p.Gly1204%3D	synonymous_variant	1	0.002557545	0
DCM & HCM	MYH7	c.3351G>A	p.Glu1117%3D	synonymous_variant	1	0.002557545	0.003448
DCM & HCM	MYH7	c.3153G>A	p.Ala1051%3D	synonymous_variant	2	0.00511509	0.008878
DCM & HCM	MYH7	c.3036C>T	p.Ala1012%3D	synonymous_variant	1	0.002557545	0.008234
DCM & HCM	MYH7	c.2967T>C	p.Ile989%3D	synonymous_variant	265	0.677749361	0.70449
DCM & HCM	MYH7	c.2655T>C	p.Asn885%3D	synonymous_variant	1	0.002557545	0
DCM & HCM	MYH7	c.2637C>T	p.Ser879%3D	synonymous_variant	3	0.007672634	0
DCM & HCM	MYH7	c.2121A>G	p.Lys707%3D	synonymous_variant	1	0.002557545	0
DCM & HCM	MYH7	c.2091T>C	p.Gly697%3D	synonymous_variant	1	0.002557545	0.00016
DCM & HCM	MYH7	c.1767C>T	p.Asn589%3D	synonymous_variant	5	0.012787724	0.038453
DCM & HCM	MYH7	c.1755C>A	p.Ile585%3D	synonymous_variant	4	0.010230179	0.00029
DCM & HCM	MYH7	c.1605A>G	p.Glu535%3D	synonymous_variant	33	0.084398977	0.1046
DCM & HCM	MYH7	c.1362G>A	p.Gln454%3D	synonymous_variant	2	0.00511509	3.00E-06
DCM & HCM	MYH7	c.1191G>A	p.Lys397%3D	synonymous_variant	6	0.015345269	2.00E-04
DCM & HCM	MYH7	c.1128C>T	p.Asp376%3D	synonymous_variant	115	0.294117647	0.378822
DCM & HCM	MYH7	c.1095G>A	p.Lys365%3D	synonymous_variant	65	0.166240409	0.15273

DCM & HCM	MYH7	c.1062C>T	p.Gly354%3D	synonymous_variant	51	0.130434783	0.096833
DCM & HCM	MYH7	c.975C>T	p.Asp325%3D	synonymous_variant	23	0.058823529	0.027138
DCM & HCM	MYH7	c.597A>G	p.Ala199%3D	synonymous_variant	18	0.046035806	0.015144
DCM & HCM	MYH7	c.354G>A	p.Ser118%3D	synonymous_variant	1	0.002557545	4.40E-05
DCM & HCM	MYH7	c.261C>T	p.Ile87%3D	synonymous_variant	1	0.002557545	0.002072
DCM & HCM	MYH7	c.210C>T	p.Thr70%3D	synonymous_variant	2	0.00511509	2.80E-05
DCM & HCM	MYH7	c.189C>T	p.Thr63%3D	synonymous_variant	312	0.797953964	0.615016
HCM	ACTC1	c.927T>C	p.Pro309%3D	synonymous_variant	29	0.074168798	0.07161
HCM	ACTC1	c.399C>T	p.Ala133%3D	synonymous_variant	4	0.010230179	5.60E-05
HCM	ACTC1	c.219C>T	p.Ile73%3D	synonymous_variant	1	0.002557545	0.000473
DCM & HCM	TPM1	c.87G>A	p.Lys29%3D	synonymous_variant	1	0.002557545	0
DCM & HCM	TPM1	c.180C>T	p.Tyr60%3D	synonymous_variant	3	0.007672634	0.008027
DCM & HCM	TPM1	c.453C>A	p.Ala151%3D	synonymous_variant	353	0.902813299	0.946449
DCM & HCM	TPM1	c.486T>C	p.Tyr162%3D	synonymous_variant	73	0.186700767	0.070244
DCM & HCM	TPM1	c.597T>C	p.Thr199%3D	synonymous_variant	1	0.002557545	0
DCM	TCAP	c.132C>T	p.Asp44%3D	synonymous_variant	1	0.002557545	0.000122

DCM	TCAP	c.453A>C	p.Ala151%3D	synonymous_variant	329	0.841432225	0.734078
syndromic gene (associated with isolated LVH)	TTR	c.333A>G	p.Ala111%3D	synonymous_variant	1	0.002557545	0
HCM	TNNI3	c.537G>A	p.Glu179%3D	synonymous_variant	105	0.268542199	0.102937
HCM	TNNI3	c.204G>T	p.Arg68%3D	synonymous_variant	26	0.066496164	0.055561
HCM	TNNI3	c.198G>A	p.Glu66%3D	synonymous_variant	8	0.020460358	0.107608
syndromic gene (associated with isolated LVH)	DES	c.75A>G	p.Pro25%3D	synonymous_variant	388	0.992327366	0.986892
syndromic gene (associated with isolated LVH)	DES	c.93T>C	p.Ser31%3D	synonymous_variant	386	0.987212276	0.987198
syndromic gene (associated with isolated LVH)	DES	c.324G>A	p.Glu108%3D	synonymous_variant	5	0.012787724	0.004611
syndromic gene (associated with isolated LVH)	DES	c.372G>A	p.Glu124%3D	synonymous_variant	30	0.076726343	0.052875
syndromic gene (associated with isolated LVH)	DES	c.408C>T	p.Leu136%3D	synonymous_variant	26	0.066496164	0.037492
syndromic gene (associated with isolated LVH)	DES	c.669T>C	p.Ile223%3D	synonymous_variant	13	0.033248082	0.087196
syndromic gene (associated with isolated LVH)	DES	c.828C>T	p.Asp276%3D	synonymous_variant	278	0.710997442	0.432104

syndromic gene (associated with isolated LVH)	DES	c.936C>T	p.Asp312%3D	synonymous_variant	1	0.002557545	2.10E-05
syndromic gene (associated with isolated LVH)	DES	c.1014G>C	p.Leu338%3D	synonymous_variant	278	0.710997442	0.43849
syndromic gene (associated with isolated LVH)	DES	c.1104G>A	p.Ala368%3D	synonymous_variant	278	0.710997442	0.431078
syndromic gene (associated with isolated LVH)	DES	c.1251T>C	p.Asn417%3D	synonymous_variant	1	0.002557545	0
syndromic gene (associated with isolated LVH)	DES	c.1269C>T	p.Tyr423%3D	synonymous_variant	1	0.002557545	2.60E-05
DCM	TTN	c.107724T>C	p.Ile35908%3D	synonymous_variant	2	0.00511509	0
DCM	TTN	c.107700A>G	p.Glu35900%3D	synonymous_variant	1	0.002557545	0.020489
DCM	TTN	c.107265T>C	p.Asn35755%3D	synonymous_variant	1	0.002557545	0
DCM	TTN	c.107202A>G	p.Glu35734%3 D	synonymous_variant	1	0.002557545	0
DCM	TTN	c.106638G>A	p.Arg35546%3 D	synonymous_variant	1	0.002557545	0.021955
DCM	TTN	c.106578T>A	p.Ser35526%3 D	synonymous_variant	7	0.017902813	0.000796
DCM	TTN	c.106476T>C	p.Cys35492%3D	synonymous_variant	5	0.012787724	0.049455
DCM	TTN	c.106275G>C	p.Gly35425%3D	synonymous_variant	73	0.186700767	0.08739
DCM	TTN	c.105582C>T	p.Ser35194%3D	synonymous_variant	74	0.189258312	0.171335

DCM	TTN	c.105468G>A	p.Pro35156%3D	synonymous_variant	2	0.00511509	0.010872
DCM	TTN	c.105453C>T	p.Asp35151%3D	synonymous_variant	1	0.002557545	0
DCM	TTN	c.105384A>G	p.Ala35128%3D	synonymous_variant	189	0.483375959	0.692318
DCM	TTN	c.105228G>A	p.Ser35076%3D	synonymous_variant	24	0.061381074	0.000925
DCM	TTN	c.104988C>T	p.Val34996%3D	synonymous_variant	95	0.242966752	0.421898
DCM	TTN	c.104922A>G	p.Lys34974%3D	synonymous_variant	1	0.002557545	1.10E-05
DCM	TTN	c.104769A>C	p.Thr34923%3D	synonymous_variant	1	0.002557545	0.00788
DCM	TTN	c.104526C>T	p.Ser34842%3D	synonymous_variant	1	0.002557545	0
DCM	TTN	c.104457C>T	p.Tyr34819%3D	synonymous_variant	1	0.002557545	0.00013
DCM	TTN	c.103302T>C	p.Tyr34434%3D	synonymous_variant	2	0.00511509	0.000157
DCM	TTN	c.102519C>T	p.Gly34173%3D	synonymous_variant	95	0.242966752	0.421988
DCM	TTN	c.101763C>T	p.His33921%3D	synonymous_variant	1	0.002557545	0
DCM	TTN	c.101406C>G	p.Val33802%3D	synonymous_variant	1	0.002557545	0.020364
DCM	TTN	c.101037G>A	p.Gln33679%3D	synonymous_variant	3	0.007672634	2.30E-05
DCM	TTN	c.100059T>A	p.Ile33353%3D	synonymous_variant	1	0.002557545	0.020339
DCM	TTN	c.98595A>G	p.Glu32865%3D	synonymous_variant	1	0.002557545	0.016873

DCM	TTN	c.98499C>T	p.Leu32833%3D	synonymous_variant	1	0.002557545	0.020363
DCM	TTN	c.98469T>C	p.Asp32823%3D	synonymous_variant	3	0.007672634	0
DCM	TTN	c.98346C>T	p.Gly32782%3D	synonymous_variant	1	0.002557545	0
DCM	TTN	c.97386C>T	p.Thr32462%3D	synonymous_variant	6	0.015345269	0.000553
DCM	TTN	c.96918C>T	p.Ile32306%3D	synonymous_variant	3	0.007672634	0.000874
DCM	TTN	c.96756T>C	p.Val32252%3D	synonymous_variant	1	0.002557545	0
DCM	TTN	c.96501T>C	p.Ser32167%3D	synonymous_variant	9	0.023017903	0.007227
DCM	TTN	c.96462A>G	p.Thr32154%3D	synonymous_variant	1	0.002557545	0
DCM	TTN	c.96189C>T	p.Thr32063%3D	synonymous_variant	1	0.002557545	5.20E-05
DCM	TTN	c.95613C>T	p.Thr31871%3D	synonymous_variant	1	0.002557545	0
DCM	TTN	c.95553C>T	p.Ser31851%3D	synonymous_variant	4	0.010230179	0.063085
DCM	TTN	c.95205C>T	p.Asp31735%3D	synonymous_variant	1	0.002557545	0.001671
DCM	TTN	c.95148C>T	p.Thr31716%3D	synonymous_variant	1	0.002557545	0.01974
DCM	TTN	c.94863C>T	p.His31621%3D	synonymous_variant	14	0.035805627	0.000591
DCM	TTN	c.94464T>C	p.Ala31488%3D	synonymous_variant	1	0.002557545	0.000699
DCM	TTN	c.94239C>A	p.Thr31413%3D	synonymous_variant	1	0.002557545	0

DCM	TTN	c.94053G>A	p.Ser31351%3D	synonymous_variant	1	0.002557545	2.60E-05
DCM	TTN	c.93972A>G	p.Glu31324%3D	synonymous_variant	2	0.00511509	7.00E-06
DCM	TTN	c.93900C>T	p.Ser31300%3D	synonymous_variant	1	0.002557545	0.001066
DCM	TTN	c.93387C>T	p.Ser31129%3D	synonymous_variant	17	0.043478261	0.030262
DCM	TTN	c.93243C>T	p.Ala31081%3D	synonymous_variant	96	0.245524297	0.422096
DCM	TTN	c.92901C>T	p.Ser30967%3D	synonymous_variant	12	0.030690537	0.028768
DCM	TTN	c.92715C>T	p.Gly30905%3D	synonymous_variant	1	0.002557545	0.001251
DCM	TTN	c.91071T>G	p.Thr30357%3D	synonymous_variant	41	0.104859335	0.254363
DCM	TTN	c.89994G>A	p.Ser29998%3D	synonymous_variant	1	0.002557545	0.020306
DCM	TTN	c.88858C>T	p.Leu29620%3D	synonymous_variant	1	0.002557545	0.020081
DCM	TTN	c.88476C>G	p.Thr29492%3D	synonymous_variant	1	0.002557545	0.002289
DCM	TTN	c.88272G>A	p.Glu29424%3D	synonymous_variant	17	0.043478261	0.190652
DCM	TTN	c.88083C>T	p.Phe29361%3D	synonymous_variant	1	0.002557545	1.10E-05
DCM	TTN	c.87771C>A	p.Gly29257%3D	synonymous_variant	6	0.015345269	0.000168
DCM	TTN	c.87669T>C	p.His29223%3D	synonymous_variant	4	0.010230179	0.063202
DCM	TTN	c.87087T>C	p.Leu29029%3D	synonymous_variant	1	0.002557545	0.05844

DCM	TTN	c.86811A>G	p.Val28937%3D	synonymous_variant	9	0.023017903	0.030425
DCM	TTN	c.86526T>G	p.Val28842%3D	synonymous_variant	1	0.002557545	0.000481
DCM	TTN	c.86301G>A	p.Lys28767%3D	synonymous_variant	2	0.00511509	0.010834
DCM	TTN	c.84453A>G	p.Pro28151%3D	synonymous_variant	5	0.012787724	0.031494
DCM	TTN	c.83673T>C	p.Gly27891%3D	synonymous_variant	188	0.480818414	0.693865
DCM	TTN	c.82740G>A	p.Thr27580%3D	synonymous_variant	3	0.007672634	0.020018
DCM	TTN	c.82575G>A	p.Thr27525%3D	synonymous_variant	7	0.017902813	0.027591
DCM	TTN	c.81855C>T	p.Ile27285%3D	synonymous_variant	1	0.002557545	0.056276
DCM	TTN	c.81558T>C	p.Asn27186%3D	synonymous_variant	1	0.002557545	0.005354
DCM	TTN	c.81192T>C	p.Val27064%3D	synonymous_variant	1	0.002557545	0
DCM	TTN	c.80799C>A	p.Thr26933%3D	synonymous_variant	1	0.002557545	0.057826
DCM	TTN	c.80187G>A	p.Ala26729%3D	synonymous_variant	1	0.002557545	2.30E-05
DCM	TTN	c.79728A>C	p.Ser26576%3D	synonymous_variant	1	0.002557545	7.00E-06
DCM	TTN	c.79689C>A	p.Val26563%3D	synonymous_variant	3	0.007672634	0.023817
DCM	TTN	c.79062T>A	p.Gly26354%3D	synonymous_variant	74	0.189258312	0.177309
DCM	TTN	c.78147A>G	p.Gln26049%3D	synonymous_variant	1	0.002557545	0.001231

DCM	TTN	c.77556C>T	p.Leu25852%3D	synonymous_variant	1	0.002557545	0
DCM	TTN	c.77205G>A	p.Val25735%3D	synonymous_variant	3	0.007672634	0.001516
DCM	TTN	c.76854A>G	p.Val25618%3D	synonymous_variant	3	0.007672634	9.60E-05
DCM	TTN	c.76722T>C	p.Tyr25574%3D	synonymous_variant	2	0.00511509	0.00375
DCM	TTN	c.75522A>C	p.Ala25174%3D	synonymous_variant	35	0.089514066	0.213288
DCM	TTN	c.74331C>T	p.Asp24777%3D	synonymous_variant	1	0.002557545	2.30E-05
DCM	TTN	c.73914G>A	p.Glu24638%3D	synonymous_variant	1	0.002557545	3.00E-06
DCM	TTN	c.73336C>T	p.Leu24446%3D	synonymous_variant	1	0.002557545	0.002599
DCM	TTN	c.72624A>G	p.Pro24208%3D	synonymous_variant	1	0.002557545	0.020218
DCM	TTN	c.72105T>C	p.Phe24035%3D	synonymous_variant	1	0.002557545	0.012897
DCM	TTN	c.72036T>C	p.Ser24012%3D	synonymous_variant	1	0.002557545	0
DCM	TTN	c.72033A>G	p.Pro24011%3D	synonymous_variant	1	0.002557545	0.025125
DCM	TTN	c.71940G>A	p.Leu23980%3D	synonymous_variant	1	0.002557545	0.025001
DCM	TTN	c.71247C>T	p.Asp23749%3D	synonymous_variant	1	0.002557545	0
DCM	TTN	c.70983G>A	p.Pro23661%3D	synonymous_variant	1	0.002557545	2.60E-05
DCM	TTN	c.70830C>T	p.Ser23610%3D	synonymous_variant	143	0.3657289	0.299239

DCM	TTN	c.70651C>T	p.Leu23551%3D	synonymous_variant	7	0.017902813	0.000276
DCM	TTN	c.70506G>T	p.Gly23502%3D	synonymous_variant	1	0.002557545	9.50E-05
DCM	TTN	c.70194T>C	p.Thr23398%3D	synonymous_variant	1	0.002557545	0.000178
DCM	TTN	c.69045G>A	p.Ala23015%3D	synonymous_variant	1	0.002557545	1.70E-05
DCM	TTN	c.68641C>A	p.Arg22881%3D	synonymous_variant	1	0.002557545	0
DCM	TTN	c.68217T>C	p.His22739%3D	synonymous_variant	34	0.086956522	0.205147
DCM	TTN	c.68079G>A	p.Thr22693%3D	synonymous_variant	1	0.002557545	0.015001
DCM	TTN	c.67950A>C	p.Pro22650%3D	synonymous_variant	1	0.002557545	0
DCM	TTN	c.66324A>G	p.Lys22108%3D	synonymous_variant	1	0.002557545	0
DCM	TTN	c.65775C>T	p.Ser21925%3D	synonymous_variant	11	0.028132992	0.009588
DCM	TTN	c.65682A>G	p.Thr21894%3D	synonymous_variant	194	0.496163683	0.694472
DCM	TTN	c.65499A>G	p.Arg21833%3D	synonymous_variant	1	0.002557545	8.80E-05
DCM	TTN	c.65319T>C	p.Thr21773%3D	synonymous_variant	3	0.007672634	4.70E-05
DCM	TTN	c.65022C>T	p.Asp21674%3D	synonymous_variant	1	0.002557545	3.00E-06
DCM	TTN	c.64680C>T	p.Pro21560%3D	synonymous_variant	1	0.002557545	0
DCM	TTN	c.64032C>T	p.Asn21344%3D	synonymous_variant	1	0.002557545	0.032621

DCM	TTN	c.63981A>G	p.Val21327%3D	synonymous_variant	1	0.002557545	0.001916
DCM	TTN	c.63876C>T	p.Asn21292%3D	synonymous_variant	1	0.002557545	0.024905
DCM	TTN	c.62178T>C	p.Thr20726%3D	synonymous_variant	5	0.012787724	0.047663
DCM	TTN	c.62058T>C	p.Tyr20686%3D	synonymous_variant	194	0.496163683	0.700706
DCM	TTN	c.61245A>G	p.Thr20415%3D	synonymous_variant	194	0.496163683	0.700644
DCM	TTN	c.61029T>C	p.Phe20343%3D	synonymous_variant	3	0.007672634	0.003557
DCM	TTN	c.60312T>C	p.Gly20104%3D	synonymous_variant	1	0.002557545	0
DCM	TTN	c.60198G>A	p.Pro20066%3D	synonymous_variant	1	0.002557545	7.50E-05
DCM	TTN	c.60180C>A	p.Leu20060%3D	synonymous_variant	1	0.002557545	0
DCM	TTN	c.59943C>A	p.Pro19981%3D	synonymous_variant	2	0.00511509	0.01177
DCM	TTN	c.59634T>C	p.Pro19878%3D	synonymous_variant	3	0.007672634	2.30E-05
DCM	TTN	c.59439G>A	p.Val19813%3D	synonymous_variant	1	0.002557545	7.00E-06
DCM	TTN	c.59235C>T	p.Thr19745%3D	synonymous_variant	1	0.002557545	4.40E-05
DCM	TTN	c.58933C>T	p.Leu19645%3D	synonymous_variant	2	0.00511509	0.217371
DCM	TTN	c.58527C>T	p.Gly19509%3D	synonymous_variant	1	0.002557545	0
DCM	TTN	c.58419A>G	p.Gln19473%3D	synonymous_variant	2	0.00511509	0.00455

DCM	TTN	c.57648C>T	p.Ile19216%3D	synonymous_variant	1	0.002557545	0.02011
DCM	TTN	c.57462G>A	p.Gln19154%3D	synonymous_variant	3	0.007672634	0.019494
DCM	TTN	c.57315T>C	p.His19105%3D	synonymous_variant	125	0.319693095	0.302597
DCM	TTN	c.56655T>C	p.Pro18885%3D	synonymous_variant	1	0.002557545	0
DCM	TTN	c.56019T>C	p.Thr18673%3D	synonymous_variant	3	0.007672634	9.50E-05
DCM	TTN	c.55659G>A	p.Val18553%3D	synonymous_variant	1	0.002557545	0.0019
DCM	TTN	c.55515C>T	p.Asp18505%3D	synonymous_variant	3	0.007672634	2.60E-05
DCM	TTN	c.54453T>C	p.Asp18151%3D	synonymous_variant	1	0.002557545	0
DCM	TTN	c.52917T>C	p.Asp17639%3D	synonymous_variant	19	0.04859335	0.069714
DCM	TTN	c.52821T>C	p.Asp17607%3D	synonymous_variant	2	0.00511509	0.098909
DCM	TTN	c.51684G>A	p.Ala17228%3D	synonymous_variant	1	0.002557545	0.095529
DCM	TTN	c.51249C>A	p.Val17083%3D	synonymous_variant	1	0.002557545	0.000238
DCM	TTN	c.50154C>A	p.Gly16718%3D	synonymous_variant	1	0.002557545	0
DCM	TTN	c.49998T>C	p.Asn16666%3D	synonymous_variant	1	0.002557545	0.002225
DCM	TTN	c.49758T>C	p.Tyr16586%3D	synonymous_variant	1	0.002557545	0.001975
DCM	TTN	c.49731T>C	p.His16577%3D	synonymous_variant	25	0.063938619	0.129024

DCM	TTN	c.49708T>C	p.Leu16570%3D	synonymous_variant	2	0.00511509	7.00E-06
DCM	TTN	c.49371A>T	p.Leu16457%3D	synonymous_variant	1	0.002557545	0.020678
DCM	TTN	c.49152A>C	p.Thr16384%3D	synonymous_variant	1	0.002557545	0
DCM	TTN	c.48996G>A	p.Glu16332%3D	synonymous_variant	25	0.063938619	0.126158
DCM	TTN	c.47400G>A	p.Lys15800%3D	synonymous_variant	16	0.040920716	0.034223
DCM	TTN	c.47322T>C	p.Gly15774%3D	synonymous_variant	2	0.00511509	0
DCM	TTN	c.46386C>T	p.Cys15462%3D	synonymous_variant	1	0.002557545	4.60E-05
DCM	TTN	c.45994C>T	p.Leu15332%3D	synonymous_variant	1	0.002557545	3.90E-05
DCM	TTN	c.45738T>C	p.Ala15246%3D	synonymous_variant	2	0.00511509	0.099238
DCM	TTN	c.45526C>T	p.Leu15176%3D	synonymous_variant	24	0.061381074	0.077442
DCM	TTN	c.45174C>T	p.Gly15058%3D	synonymous_variant	2	0.00511509	0.002297
DCM	TTN	c.44907T>C	p.Asn14969%3D	synonymous_variant	1	0.002557545	0
DCM	TTN	c.44784T>C	p.Asp14928%3D	synonymous_variant	1	0.002557545	0.002012
DCM	TTN	c.43596T>C	p.Asn14532%3D	synonymous_variant	10	0.025575448	0.120451
DCM	TTN	c.43488G>A	p.Arg14496%3D	synonymous_variant	3	0.007672634	0.029024
DCM	TTN	c.42978C>T	p.Tyr14326%3D	synonymous_variant	2	0.00511509	0.005409

DCM	TTN	c.42918C>T	p.Asp14306%3D	synonymous_variant	1	0.002557545	0.000276
DCM	TTN	c.42783A>G	p.Lys14261%3D	synonymous_variant	20	0.051150895	0.190016
DCM	TTN	c.42156C>T	p.Ile14052%3D	synonymous_variant	1	0.002557545	0.020629
DCM	TTN	c.41508T>C	p.Ala13836%3D	synonymous_variant	2	0.00511509	0.099096
DCM	TTN	c.41166C>T	p.Asp13722%3D	synonymous_variant	1	0.002557545	0.001504
DCM	TTN	c.41103C>T	p.Gly13701%3D	synonymous_variant	6	0.015345269	0.003965
DCM	TTN	c.41097C>T	p.Phe13699%3D	synonymous_variant	1	0.002557545	3.50E-05
DCM	TTN	c.40587A>G	p.Glu13529%3D	synonymous_variant	2	0.00511509	0.004353
DCM	TTN	c.40164A>C	p.Ile13388%3D	synonymous_variant	1	0.002557545	0
DCM	TTN	c.40068G>A	p.Val13356%3D	synonymous_variant	1	0.002557545	0
DCM	TTN	c.39786A>G	p.Glu13262%3D	synonymous_variant	2	0.00511509	9.60E-05
DCM	TTN	c.38880A>G	p.Pro12960%3D	synonymous_variant	2	0.00511509	0.096926
DCM	TTN	c.38835A>G	p.Pro12945%3D	synonymous_variant	1	0.002557545	0
DCM	TTN	c.36489G>A	p.Ala12163%3D	synonymous_variant	13	0.033248082	0.034901
DCM	TTN	c.36390C>T	p.Ile12130%3D	synonymous_variant	1	0.002557545	5.60E-05
DCM	TTN	c.36318A>G	p.Lys12106%3D	synonymous_variant	33	0.084398977	0.060297

DCM	TTN	c.34734A>G	p.Val11578%3D	synonymous_variant	2	0.00511509	0
DCM	TTN	c.33834G>A	p.Glu11278%3D	synonymous_variant	147	0.375959079	0.429124
DCM	TTN	c.33063A>G	p.Glu11021%3D	synonymous_variant	2	0.00511509	0.008513
DCM	TTN	c.32367G>A	p.Lys10789%3D	synonymous_variant	2	0.00511509	0.006646
DCM	TTN	c.32253A>C	p.Ser10751%3D	synonymous_variant	1	0.002557545	0
DCM	TTN	c.31830A>G	p.Glu10610%3D	synonymous_variant	1	0.002557545	0
DCM	TTN	c.31752T>C	p.Ala10584%3D	synonymous_variant	1	0.002557545	4.40E-05
DCM	TTN	c.30384T>C	p.Asp10128%3D	synonymous_variant	1	0.002557545	0.020489
DCM	TTN	c.30231A>G	p.Pro10077%3D	synonymous_variant	7	0.017902813	0.009028
DCM	TTN	c.29799G>A	p.Ser9933%3D	synonymous_variant	17	0.043478261	0.203064
DCM	TTN	c.29763T>C	p.Ile9921%3D	synonymous_variant	22	0.056265985	0.204495
DCM	TTN	c.29541C>T	p.Phe9847%3D	synonymous_variant	2	0.00511509	0.003555
DCM	TTN	c.28983G>A	p.Val9661%3D	synonymous_variant	2	0.00511509	0
DCM	TTN	c.28980T>C	p.Tyr9660%3D	synonymous_variant	1	0.002557545	0
DCM	TTN	c.28662G>A	p.Arg9554%3D	synonymous_variant	13	0.033248082	0.205258
DCM	TTN	c.28131C>T	p.Asn9377%3D	synonymous_variant	2	0.00511509	0.000515

DCM	TTN	c.26991A>G	p.Thr8997%3D	synonymous_variant	20	0.051150895	0.070489
DCM	TTN	c.26928G>A	p.Leu8976%3D	synonymous_variant	1	0.002557545	0.000127
DCM	TTN	c.26694G>T	p.Gly8898%3D	synonymous_variant	1	0.002557545	0.001073
DCM	TTN	c.26682G>A	p.Pro8894%3D	synonymous_variant	5	0.012787724	0.016657
DCM	TTN	c.26655C>T	p.Ser8885%3D	synonymous_variant	171	0.437340153	0.65798
DCM	TTN	c.26466C>G	p.Ala8822%3D	synonymous_variant	1	0.002557545	0.008069
DCM	TTN	c.26289A>G	p.Glu8763%3D	synonymous_variant	178	0.455242967	0.660006
DCM	TTN	c.26223G>A	p.Lys8741%3D	synonymous_variant	1	0.002557545	0
DCM	TTN	c.26091A>T	p.Leu8697%3D	synonymous_variant	171	0.437340153	0.659736
DCM	TTN	c.25707T>C	p.Tyr8569%3D	synonymous_variant	1	0.002557545	0.203951
DCM	TTN	c.25347C>T	p.Leu8449%3D	synonymous_variant	1	0.002557545	0
DCM	TTN	c.25008C>T	p.Cys8336%3D	synonymous_variant	15	0.038363171	0.011757
DCM	TTN	c.24909G>A	p.Lys8303%3D	synonymous_variant	10	0.025575448	0.035877
DCM	TTN	c.24579A>G	p.Thr8193%3D	synonymous_variant	2	0.00511509	0.0214
DCM	TTN	c.24516C>T	p.Thr8172%3D	synonymous_variant	54	0.138107417	0.107373
DCM	TTN	c.24471C>T	p.Gly8157%3D	synonymous_variant	2	0.00511509	0.014262

DCM	TTN	c.24345C>T	p.Ser8115%3D	synonymous_variant	2	0.00511509	0.021409
DCM	TTN	c.24150C>T	p.Ser8050%3D	synonymous_variant	1	0.002557545	0.020459
DCM	TTN	c.24123T>G	p.Thr8041%3D	synonymous_variant	1	0.002557545	0
DCM	TTN	c.23853C>A	p.Ala7951%3D	synonymous_variant	10	0.025575448	0.013148
DCM	TTN	c.23646C>G	p.Val7882%3D	synonymous_variant	2	0.00511509	0
DCM	TTN	c.23223G>A	p.Gln7741%3D	synonymous_variant	391	1	0.994646
DCM	TTN	c.23215C>A	p.Arg7739%3D	synonymous_variant	1	0.002557545	3.00E-06
DCM	TTN	c.23001G>A	p.Thr7667%3D	synonymous_variant	12	0.030690537	0.03953
DCM	TTN	c.22692A>T	p.Thr7564%3D	synonymous_variant	1	0.002557545	0.003092
DCM	TTN	c.22611T>C	p.His7537%3D	synonymous_variant	8	0.020460358	0.052865
DCM	TTN	c.22080T>C	p.Asp7360%3D	synonymous_variant	32	0.081841432	0.184711
DCM	TTN	c.21489C>G	p.Thr7163%3D	synonymous_variant	1	0.002557545	0.001342
DCM	TTN	c.21148C>T	p.Leu7050%3D	synonymous_variant	2	0.00511509	0.000478
DCM	TTN	c.18903C>T	p.Thr6301%3D	synonymous_variant	2	0.00511509	0.177153
DCM	TTN	c.18831C>A	p.Gly6277%3D	synonymous_variant	1	0.002557545	0
DCM	TTN	c.18561G>A	p.Ala6187%3D	synonymous_variant	2	0.00511509	0

DCM	TTN	c.18390A>T	p.Thr6130%3D	synonymous_variant	5	0.012787724	0.043902
DCM	TTN	c.16878C>T	p.His5626%3D	synonymous_variant	2	0.00511509	0
DCM	TTN	c.16095C>T	p.Asn5365%3D	synonymous_variant	25	0.063938619	0.031442
DCM	TTN	c.15993C>T	p.Tyr5331%3D	synonymous_variant	1	0.002557545	0
DCM	TTN	c.15870G>A	p.Leu5290%3D	synonymous_variant	3	0.007672634	0
DCM	TTN	c.15861G>A	p.Thr5287%3D	synonymous_variant	1	0.002557545	2.30E-05
DCM	TTN	c.15792T>C	p.Ile5264%3D	synonymous_variant	80	0.204603581	0.078223
DCM	TTN	c.15717G>A	p.Thr5239%3D	synonymous_variant	18	0.046035806	0.06499
DCM	TTN	c.15633C>T	p.Asp5211%3D	synonymous_variant	2	0.00511509	2.60E-05
DCM	TTN	c.15309A>G	p.Pro5103%3D	synonymous_variant	1	0.002557545	0
DCM	TTN	c.14610C>T	p.Ser4870%3D	synonymous_variant	391	1	0.994661
DCM	TTN	c.14535C>T	p.Asp4845%3D	synonymous_variant	1	0.002557545	0.000313
DCM	TTN	c.13782G>A	p.Gln4594%3D	synonymous_variant	1	0.002557545	5.70E-05
DCM	TTN	c.13218C>T	p.Ala4406%3D	synonymous_variant	5	0.012787724	0.236867
DCM	TTN	c.12780G>T	p.Ala4260%3D	synonymous_variant	4	0.010230179	0.236186
DCM	TTN	c.12780G>A	p.Ala4260%3D	synonymous_variant	116	0.296675192	0.453767

DCM	TTN	c.12558A>G	p.Pro4186%3D	synonymous_variant	1	0.002557545	3.50E-05
DCM	TTN	c.12255T>C	p.Ile4085%3D	synonymous_variant	4	0.010230179	0.236835
DCM	TTN	c.12234C>G	p.Thr4078%3D	synonymous_variant	2	0.00511509	0.000705
DCM	TTN	c.12117C>T	p.Pro4039%3D	synonymous_variant	17	0.043478261	0.064053
DCM	TTN	c.11019C>T	p.Cys3673%3D	synonymous_variant	4	0.010230179	0.015969
DCM	TTN	c.10242C>T	p.Tyr3414%3D	synonymous_variant	4	0.010230179	0.007897
DCM	TTN	c.10188A>G	p.Glu3396%3D	synonymous_variant	2	0.00511509	0.000705
DCM	TTN	c.10050G>A	p.Pro3350%3D	synonymous_variant	1	0.002557545	5.00E-05
DCM	TTN	c.9879A>G	p.Glu3293%3D	synonymous_variant	391	1	0.994901
DCM	TTN	c.9789C>T	p.Ser3263%3D	synonymous_variant	1	0.002557545	0.00052
DCM	TTN	c.9597A>G	p.Glu3199%3D	synonymous_variant	29	0.074168798	0.419882
DCM	TTN	c.9150A>G	p.Thr3050%3D	synonymous_variant	1	0.002557545	0.008628
DCM	TTN	c.8937C>T	p.Asn2979%3D	synonymous_variant	1	0.002557545	7.00E-06
DCM	TTN	c.8919C>G	p.Ser2973%3D	synonymous_variant	24	0.061381074	0.181525
DCM	TTN	c.8589A>G	p.Glu2863%3D	synonymous_variant	2	0.00511509	0.00016
DCM	TTN	c.8373C>T	p.His2791%3D	synonymous_variant	1	0.002557545	0

DCM	TTN	c.7545C>T	p.Tyr2515%3D	synonymous_variant	59	0.150895141	0.110581
DCM	TTN	c.7338C>T	p.Asp2446%3D	synonymous_variant	1	0.002557545	0.000226
DCM	TTN	c.7173C>T	p.Asp2391%3D	synonymous_variant	1	0.002557545	7.50E-05
DCM	TTN	c.5697C>T	p.Ile1899%3D	synonymous_variant	1	0.002557545	0.000336
DCM	TTN	c.5388T>C	p.Asp1796%3D	synonymous_variant	1	0.002557545	0.007195
DCM	TTN	c.4347A>G	p.Ser1449%3D	synonymous_variant	4	0.010230179	0.0362
DCM	TTN	c.4197G>A	p.Val1399%3D	synonymous_variant	1	0.002557545	0
DCM	TTN	c.4170G>A	p.Pro1390%3D	synonymous_variant	1	0.002557545	5.70E-05
DCM	TTN	c.3993C>T	p.Ile1331%3D	synonymous_variant	4	0.010230179	0.044117
DCM	TTN	c.3759A>G	p.Arg1253%3D	synonymous_variant	4	0.010230179	0.235069
DCM	TTN	c.3087T>C	p.Tyr1029%3D	synonymous_variant	4	0.010230179	0.236379
DCM	TTN	c.2949C>T	p.Ile983%3D	synonymous_variant	5	0.012787724	0.041349
DCM	TTN	c.2781A>C	p.Thr927%3D	synonymous_variant	4	0.010230179	0.051863
DCM	TTN	c.2244G>A	p.Glu748%3D	synonymous_variant	209	0.534526854	0.40034
DCM	TTN	c.1551T>C	p.Thr517%3D	synonymous_variant	1	0.002557545	0
DCM	TTN	c.1368T>C	p.Thr456%3D	synonymous_variant	2	0.00511509	7.00E-06

DCM	TTN	c.1137A>G	p.Arg379%3D	synonymous_variant	2	0.00511509	0.021492
DCM	TTN	c.1002C>T	p.Thr334%3D	synonymous_variant	3	0.007672634	0.003219
DCM	TTN	c.918C>T	p.Ser306%3D	synonymous_variant	1	0.002557545	1.90E-05
DCM	TTN	c.426C>T	p.Ala142%3D	synonymous_variant	2	0.00511509	0.021494
DCM	TTN	c.204C>T	p.Pro68%3D	synonymous_variant	1	0.002557545	7.40E-05
DCM	TTN	c.180T>C	p.Asp60%3D	synonymous_variant	3	0.007672634	0.000715
DCM	TTN	c.156C>T	p.Pro52%3D	synonymous_variant	2	0.00511509	0.000473
DCM	TTN	c.21G>A	p.Thr7%3D	synonymous_variant	1	0.002557545	3.00E-06
HCM	MYL3	c.231G>C	p.Gly77%3D	synonymous_variant	1	0.002557545	0
HCM	MYL3	c.81T>C	p.Pro27%3D	synonymous_variant	1	0.002557545	0.004221
HCM	MYL3	c.69C>T	p.Pro23%3D	synonymous_variant	8	0.020460358	0.089884
syndromic gene (associated with isolated LVH)	RAF1	c.1755A>G	p.Val585%3D	synonymous_variant	8	0.020460358	0.065066
syndromic gene (associated with isolated LVH)	RAF1	c.231C>T	p.Ser77%3D	synonymous_variant	1	0.002557545	0
syndromic gene (associated with isolated LVH)	RAF1	c.30G>A	p.Thr10%3D	synonymous_variant	1	0.002557545	0
DCM	SCN5A	c.5949C>T	p.Ala1983%3D	synonymous_variant	1	0.002557545	5.90E-05

DCM	SCN5A	c.5844C>T	p.Ile1948%3D	synonymous_variant	27	0.069053708	0.211186
DCM	SCN5A	c.5712G>A	p.Ser1904%3D	synonymous_variant	1	0.002557545	0.004237
DCM	SCN5A	c.5457T>C	p.Asp1819%3D	synonymous_variant	307	0.78516624	0.643577
DCM	SCN5A	c.5175G>A	p.Pro1725%3D	synonymous_variant	1	0.002557545	4.40E-05
DCM	SCN5A	c.4848C>T	p.Phe1616%3D	synonymous_variant	10	0.025575448	0.166907
DCM	SCN5A	c.4509C>T	p.Ser1503%3D	synonymous_variant	2	0.00511509	0.004221
DCM	SCN5A	c.4218G>A	p.Gly1406%3D	synonymous_variant	1	0.002557545	0.012798
DCM	SCN5A	c.3798C>T	p.Tyr1266%3D	synonymous_variant	1	0.002557545	1.70E-05
DCM	SCN5A	c.3300G>A	p.Ala1100%3D	synonymous_variant	2	0.00511509	0
DCM	SCN5A	c.3249C>T	p.Ser1083%3D	synonymous_variant	2	0.00511509	0.005234
DCM	SCN5A	c.3195C>T	p.Asn1065%3D	synonymous_variant	1	0.002557545	0
DCM	SCN5A	c.3183A>G	p.Glu1061%3D	synonymous_variant	382	0.976982097	0.987041
DCM	SCN5A	c.2976C>T	p.Pro992%3D	synonymous_variant	1	0.002557545	0.000468
DCM	SCN5A	c.1743G>A	p.Ser581%3D	synonymous_variant	3	0.007672634	0.016169
DCM	SCN5A	c.1734C>T	p.Pro578%3D	synonymous_variant	3	0.007672634	0.000212
DCM	SCN5A	c.1587T>C	p.Ile529%3D	synonymous_variant	9	0.023017903	0.006805

DCM	SCN5A	c.1302C>T	p.Phe434%3D	synonymous_variant	5	0.012787724	0.041335
DCM	SCN5A	c.891C>T	p.Asp297%3D	synonymous_variant	1	0.002557545	2.20E-05
DCM	SCN5A	c.867C>T	p.Gly289%3D	synonymous_variant	1	0.002557545	0
DCM	SCN5A	c.744G>A	p.Lys248%3D	synonymous_variant	2	0.00511509	1.20E-05
DCM	SCN5A	c.717C>T	p.Ile239%3D	synonymous_variant	1	0.002557545	0.005001
DCM	SCN5A	c.486C>T	p.Tyr162%3D	synonymous_variant	2	0.00511509	0.008318
DCM	SCN5A	c.402C>T	p.Asn134%3D	synonymous_variant	1	0.002557545	0
DCM	SCN5A	c.354C>T	p.His118%3D	synonymous_variant	2	0.00511509	0.016604
DCM	SCN5A	c.87A>G	p.Ala29%3D	synonymous_variant	365	0.933503836	0.884624
DCM	DSP	c.21C>T	p.Ser7%3D	synonymous_variant	1	0.002557545	9.60E-05
DCM	DSP	c.105G>A	p.Gly35%3D	synonymous_variant	1	0.002557545	0.008834
DCM	DSP	c.126T>C	p.Tyr42%3D	synonymous_variant	75	0.191815857	0.101429
DCM	DSP	c.576G>A	p.Leu192%3D	synonymous_variant	1	0.002557545	0
DCM	DSP	c.741T>G	p.Ala247%3D	synonymous_variant	391	1	0.993723
DCM	DSP	c.1512T>C	p.Leu504%3D	synonymous_variant	1	0.002557545	0
DCM	DSP	c.2052C>A	p.Gly684%3D	synonymous_variant	1	0.002557545	0

DCM	DSP	c.2091A>G	p.Gly697%3D	synonymous_variant	378	0.966751918	0.796193
DCM	DSP	c.2862C>T	p.Cys954%3D	synonymous_variant	137	0.350383632	0.365402
DCM	DSP	c.3510G>A	p.Glu1170%3D	synonymous_variant	4	0.010230179	0.030726
DCM	DSP	c.3723A>G	p.Arg1241%3D	synonymous_variant	1	0.002557545	0
DCM	DSP	c.3762C>G	p.Val1254%3D	synonymous_variant	1	0.002557545	0
DCM	DSP	c.3963G>A	p.Gln1321%3D	synonymous_variant	15	0.038363171	0.017246
DCM	DSP	c.4161T>C	p.Asp1387%3D	synonymous_variant	1	0.002557545	0
DCM	DSP	c.4515G>A	p.Ala1505%3D	synonymous_variant	1	0.002557545	1.70E-05
DCM	DSP	c.4773G>A	p.Arg1591%3D	synonymous_variant	1	0.002557545	0.018296
DCM	DSP	c.5304G>C	p.Gly1768%3D	synonymous_variant	1	0.002557545	0.002547
DCM	DSP	c.6192T>C	p.Asn2064%3D	synonymous_variant	1	0.002557545	0.000162
DCM	DSP	c.6390T>C	p.Ala2130%3D	synonymous_variant	4	0.010230179	0.013867
DCM	DSP	c.7122C>T	p.Thr2374%3D	synonymous_variant	240	0.613810742	0.353304
DCM	DSP	c.7995G>A	p.Thr2665%3D	synonymous_variant	2	0.00511509	0.007944
DCM	DSP	c.8175C>A	p.Arg2725%3D	synonymous_variant	22	0.056265985	0.066039
DCM	DSP	c.8301C>G	p.Thr2767%3D	synonymous_variant	3	0.007672634	0.000649

DCM	DSP	c.8415C>T	p.Ala2805%3D	synonymous_variant	7	0.017902813	0.000427
DCM	DSP	c.8472G>C	p.Gly2824%3D	synonymous_variant	368	0.941176471	0.792947
syndromic gene (associated with isolated LVH)	PRKAG2	c.1704G>A	p.Thr568%3D	synonymous_variant	1	0.002557545	0.003718
syndromic gene (associated with isolated LVH)	PRKAG2	c.1623T>C	p.Ile541%3D	synonymous_variant	17	0.043478261	0.124959
syndromic gene (associated with isolated LVH)	PRKAG2	c.1593G>A	p.Arg531%3D	synonymous_variant	2	0.00511509	0.005359
syndromic gene (associated with isolated LVH)	PRKAG2	c.1296G>A	p.Thr432%3D	synonymous_variant	1	0.002557545	0.008081
syndromic gene (associated with isolated LVH)	PRKAG2	c.1098A>G	p.Pro366%3D	synonymous_variant	3	0.007672634	0.005067
syndromic gene (associated with isolated LVH)	PRKAG2	c.981A>G	p.Leu327%3D	synonymous_variant	3	0.007672634	7.50E-05
syndromic gene (associated with isolated LVH)	PRKAG2	c.639C>T	p.Thr213%3D	synonymous_variant	10	0.025575448	0.01429
syndromic gene (associated with isolated LVH)	PRKAG2	c.123C>T	p.Ser41%3D	synonymous_variant	2	0.00511509	0.00011
syndromic gene (associated with isolated LVH)	PRKAG2	c.111T>A	p.Ile37%3D	synonymous_variant	1	0.002557545	0.003668

syndromic gene (associated with isolated LVH)	FHL1	c.6G>A	p.Ala2%3D	synonymous_variant	1	0.002557545	0
syndromic gene (associated with isolated LVH)	FHL1	c.804G>A	p.Gln268%3D	synonymous_variant	1	0.002557545	0
syndromic gene (associated with isolated LVH)	LAMP2	c.1116C>T	p.Asp372%3D	synonymous_variant	1	0.002557545	0
syndromic gene (associated with isolated LVH)	LAMP2	c.591G>A	p.Val197%3D	synonymous_variant	3	0.007672634	0.000171
syndromic gene (associated with isolated LVH)	LAMP2	c.156A>T	p.Val52%3D	synonymous_variant	211	0.539641944	0.407859

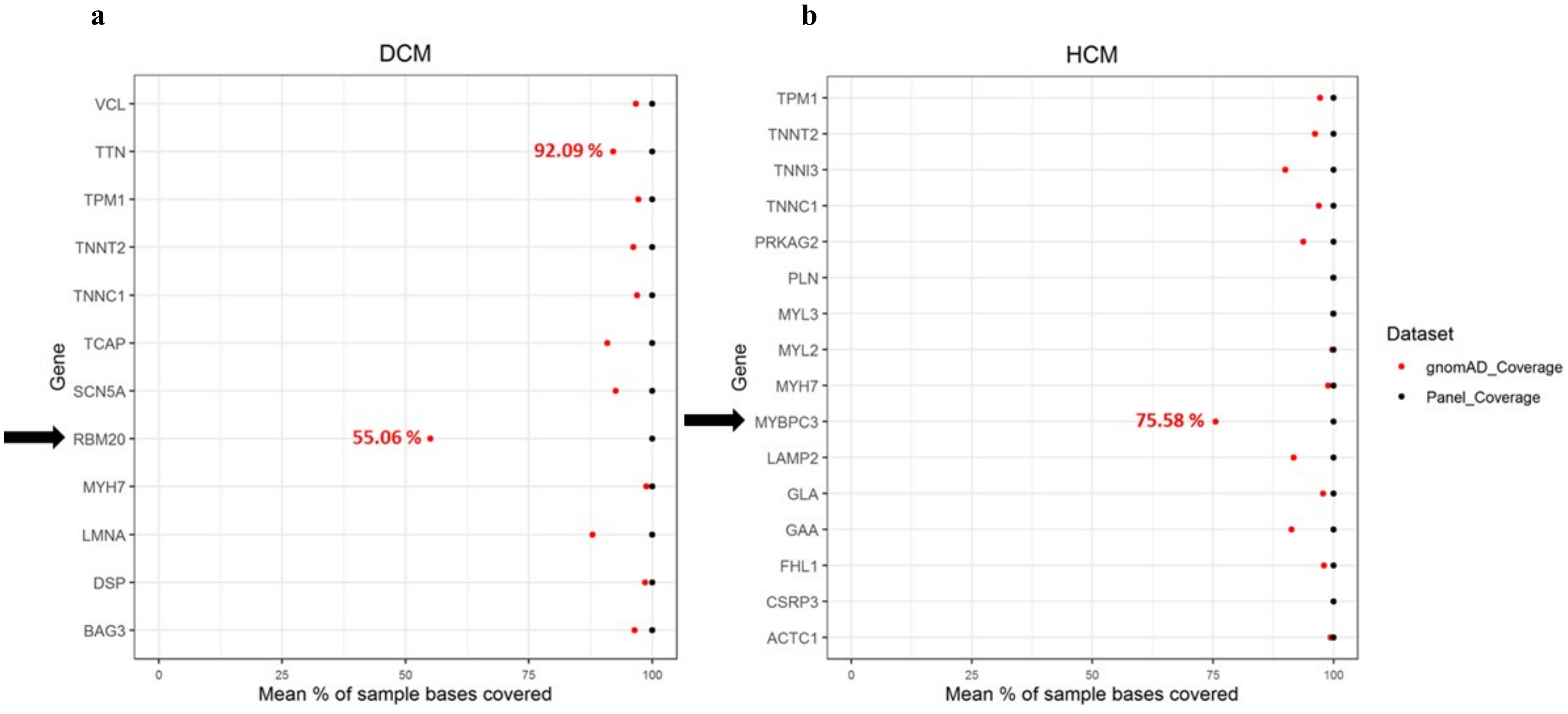

Supplementary Figure 1: Comparison of sequencing coverage in DCM/HCM genes between EHVol (ICC Panel) and gnomAD version 2.1 data (WES). Sequencing coverage differences between EHVol and gnomAD for: (a) the 12 selected DCM genes and (b) the 16 selected HCM genes. The x-axis represents the percentage of bases covered for each gene at 20X depth of coverage. Mean percentage of sample bases covered for RBM20, TTN and MYBPC3 are indicated in red.

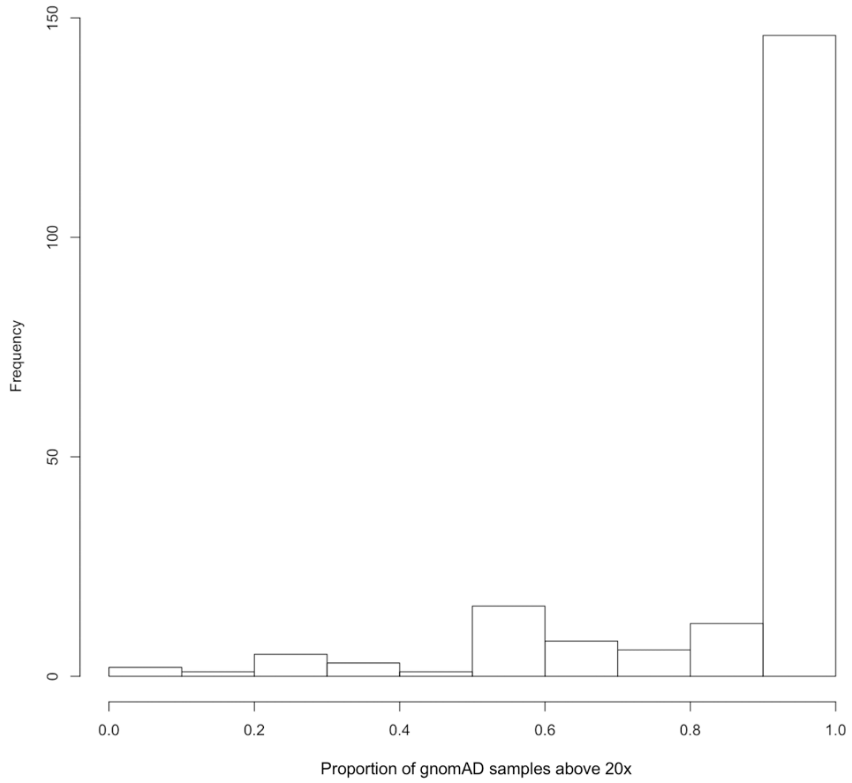

Supplementary Figure 2: Assessment of coverage in gnomAD of the positions of the 200 non-gnomAD identified in the EHVol cohort. Histogram shows the distribution of the proportion of gnomAD samples that had more than 20x average coverage for the positions of the 200 non- gnomAD variants.
