# Supplementary material for: The Egyptian Collaborative Cardiac Genomics (ECCO-GEN) Project: defining a healthy volunteer cohort
Source: NPJ Genom Med. 2020 Oct 23;5:46. doi: 10.1038/s41525-020-00153-w (PMC7584615; doi:10.1038/s41525-020-00153-w)
Supplement: Supplementary file 2 — Reporting Summary [file 41525_2020_153_MOESM2_ESM.pdf]

nature research

Corresponding author(s): Dr.Yasmine Aguib

Last updated by author(s): Aug 5, 2020

Reporting Summary

Nature Research wishes to improve the reproducibility of the work that we publish. This form provides structure for consistency and transparency in reporting. For further information on Nature Research policies, see Authors & Referees and the Editorial Policy Checklist.

Statistics

For all statistical analyses, confirm that the following items are present in the figure legend, table legend, main text, or Methods section.

n/a	Confirmed	
☐	☒	The exact sample size (*n*) for each experimental group/condition, given as a discrete number and unit of measurement
☐	☒	A statement on whether measurements were taken from distinct samples or whether the same sample was measured repeatedly
☐	☒	The statistical test(s) used AND whether they are one- or two-sided<br>*Only common tests should be described solely by name; describe more complex techniques in the Methods section.*
☒	☐	A description of all covariates tested
☒	☐	A description of any assumptions or corrections, such as tests of normality and adjustment for multiple comparisons
☐	☒	A full description of the statistical parameters including central tendency (e.g. means) or other basic estimates (e.g. regression coefficient) AND variation (e.g. standard deviation) or associated estimates of uncertainty (e.g. confidence intervals)
☐	☒	For null hypothesis testing, the test statistic (e.g. *F*, *t*, *r*) with confidence intervals, effect sizes, degrees of freedom and *P* value noted<br>*Give P values as exact values whenever suitable.*
☒	☐	For Bayesian analysis, information on the choice of priors and Markov chain Monte Carlo settings
☒	☐	For hierarchical and complex designs, identification of the appropriate level for tests and full reporting of outcomes
☒	☐	Estimates of effect sizes (e.g. Cohen's *d*, Pearson's *r*), indicating how they were calculated

Our web collection on statistics for biologists contains articles on many of the points above.

Software and code

Policy information about availability of computer code

Data collection	ICC panel data from this study was generated from the control software on the Illumina Miseq and Nextseq platforms. Any other sequencing data used is described in the Methods section.
Data analysis	For sequencing data, we used the GATK best practices workflow for germline short variant discovery and for downstream analysis and graphs, we used R. For further information on codes used, please refer to Code Availability statement.

For manuscripts utilizing custom algorithms or software that are central to the research but not yet described in published literature, software must be made available to editors/reviewers. We strongly encourage code deposition in a community repository (e.g. GitHub). See the Nature Research guidelines for submitting code & software for further information.

Data

Policy information about availability of data

All manuscripts must include a data availability statement. This statement should provide the following information, where applicable:

- Accession codes, unique identifiers, or web links for publicly available datasets
- A list of figures that have associated raw data
- A description of any restrictions on data availability

Sequencing (ICC panel) data from this study were submitted to the European Genome-phenome Archive (EGA), which is hosted by The European Bioinformatics Institute (EBI) and the Centre for Genomic Regulation (CRG) under the accession code: EGAD00001006160. All other data generated or analyzed during this study are included in this published article (and its supplementary information files).

Field-specific reporting

Please select the one below that is the best fit for your research. If you are not sure, read the appropriate sections before making your selection.

☒ Life sciences ☐ Behavioural & social sciences ☐ Ecological, evolutionary & environmental sciences

For a reference copy of the document with all sections, see nature.com/documents/nr-reporting-summary-flat.pdf

Life sciences study design

All studies must disclose on these points even when the disclosure is negative.

Sample size	Sample size reflects number of study participants fulfilling inclusion criteria until manuscript intiation date.
Data exclusions	First round of exclusion is described under 'study protocol and data collection' in the Methods section. Second round of exclusion is described under 'cardiovascular phenotyping' in the Methods section
Replication	N/A
Randomization	N/A
Blinding	N/A

Reporting for specific materials, systems and methods

We require information from authors about some types of materials, experimental systems and methods used in many studies. Here, indicate whether each material, system or method listed is relevant to your study. If you are not sure if a list item applies to your research, read the appropriate section before selecting a response.

Materials & experimental systems

n/a	Involved in the study	
☒	☐	Antibodies
☒	☐	Eukaryotic cell lines
☒	☐	Palaeontology
☒	☐	Animals and other organisms
☐	☒	Human research participants
☒	☐	Clinical data

Methods

n/a	Involved in the study	
☒	☐	ChIP-seq
☒	☐	Flow cytometry
☒	☐	MRI-based neuroimaging

Human research participants

Policy information about studies involving human research participants

Population characteristics	This is a population-based study to recruit Egyptian healthy volunteers (EHVols) from across Egypt. Refer to "Characteristics of the EHVol Study Population" in the Results section.
Recruitment	Study participants were recruited from the general population via announcements (brochures, flyers, public events) and completed a questionnaire providing demographic data, family and clinical history.
Ethics oversight	All participants provided informed consent, and the study was reviewed and approved by the local research ethics committee (20160401MYFAHC_HVOL).

Note that full information on the approval of the study protocol must also be provided in the manuscript.
